# Identification of Wnt Pathway Target Genes Regulating the Division and Differentiation of Larval Seam Cells and Vulval Precursor Cells in *Caenorhabditis elegans*

**DOI:** 10.1534/g3.115.017715

**Published:** 2015-06-05

**Authors:** Lakshmi Gorrepati, Michael W. Krause, Weiping Chen, Thomas M. Brodigan, Margarita Correa-Mendez, David M. Eisenmann

**Affiliations:** * Department of Biological Sciences, University of Maryland Baltimore County, Baltimore, Maryland 21250; † Intramural Research Program, NIDDK, Bethesda, Maryland 20814

**Keywords:** Wnt signaling, mRNA tagging, seam cells, vulval precursor cells, *C. elegans*, differentiation

## Abstract

The evolutionarily conserved Wnt/β-catenin signaling pathway plays a fundamental role during metazoan development, regulating numerous processes including cell fate specification, cell migration, and stem cell renewal. Wnt ligand binding leads to stabilization of the transcriptional effector β-catenin and upregulation of target gene expression to mediate a cellular response. During larval development of the nematode *Caenorhabditis elegans*, Wnt/β-catenin pathways act in fate specification of two hypodermal cell types, the ventral vulval precursor cells (VPCs) and the lateral seam cells. Because little is known about targets of the Wnt signaling pathways acting during larval VPC and seam cell differentiation, we sought to identify genes regulated by Wnt signaling in these two hypodermal cell types. We conditionally activated Wnt signaling in larval animals and performed cell type–specific "mRNA tagging" to enrich for VPC and seam cell–specific mRNAs, and then used microarray analysis to examine gene expression compared to control animals. Two hundred thirty-nine genes activated in response to Wnt signaling were identified, and we characterized 50 genes further. The majority of these genes are expressed in seam and/or vulval lineages during normal development, and reduction of function for nine genes caused defects in the proper division, fate specification, fate execution, or differentiation of seam cells and vulval cells. Therefore, the combination of these techniques was successful at identifying potential cell type–specific Wnt pathway target genes from a small number of cells and at increasing our knowledge of the specification and behavior of these *C. elegans* larval hypodermal cells.

The proper development of metazoans is governed by changes in the transcriptional profile of various tissues and cell types over time, and several evolutionarily conserved signaling pathways function to stimulate the transcriptional changes necessary for changes in cell fate and behavior during development. The Wnt/β-catenin signaling pathway is one highly conserved extracellular signaling pathway widely used during metazoan development; however, Wnt signaling also acts in the maintenance of tissue homeostasis via regulation of stem cell renewal (Cadigan and Peifer 2009; Schuijers and Clevers 2012). Due to its widespread use, it is not surprising that the Wnt pathway is aberrantly activated in many diseases including cancer (Polakis 2012; Clevers and Nusse 2012). The Wnt/β-catenin pathway switches between active and inactive states based on the stability of a key transcription factor β-catenin (Cadigan 2012; Clevers and Nusse 2012) . In the absence of the Wnt ligand, cytoplasmic β-catenin is bound by the components of a “destruction complex” comprising Axin, kinases CK1α and GSK3, and the adenomatous polyposis coli (APC) protein, which phosphorylates β-catenin and marks it for proteosomal degradation. The binding of the Wnt ligand to its receptor results in the disruption of the destruction complex, thus stabilizing β-catenin and allowing its entry into the nucleus where it binds to a member of the TCF/LEF transcription factor family to activate expression of Wnt pathway target genes. Microarray and ChIP-based techniques have been used in different vertebrate model systems and tissue culture to identify a spectrum of target genes activated and inhibited by Wnt signaling, providing an understanding of cellular responses as they occur *in vivo* during normal development and in the disease state (see Vlad *et al.* 2008; Nusse 2013).

As in other organisms, Wnt signaling is also utilized many times during development of the nematode *C. elegans*, where it regulates cell fate specification, polarity, and migration in the embryo and larva. Unlike other organisms, there are two β-catenin–dependent Wnt pathways in *C. elegans*, the Wnt/BAR-1 canonical (WBC) pathway and the Wnt/β-catenin asymmetry pathway (for review, see Jackson and Eisenmann 2012; Sawa and Korswagen 2013). The mechanism of the WBC pathway, which functions in several larval developmental decisions, is similar to the well-described vertebrate Wnt/β-catenin signaling pathway. However, the WBA pathway, which functions in both the embryo and larva in asymmetric divisions requiring the adoption of distinct daughter fates, has a mechanism that may be unique to *C. elegans*. In this pathway, the asymmetric distribution of key pathway components in the mother and daughter cells, along with the simultaneous activation of a pathway involving TAK-1 and NLK homologs, leads to Wnt pathway activation in one daughter of an asymmetric division but not the other, resulting in adoption of distinct daughter cell fates. Our laboratory and others have shown that Wnt signaling regulates the development of two cell types that are part of the nematode hypodermis, the single-cell-layer outer covering of the animal (Chisholm and Hsiao 2012). The WBC pathway plays a role in the specification of the larval vulval precursor cells (VPCs), which divide to generate the *C. elegans* adult vulva, whereas the WBA pathway functions in the division and specification of specialized epithelial cells called seam cells.

The VPCs are six equipotent cells that are specified during the L2/L3 molt to adopt the 1°, 2°, or 3° cell fate based on integration of information from Ras, Notch, and Wnt signaling pathways (Sternberg 2005). Three VPCs adopt 1° and 2° fates and divide to generate cells that form the vulval opening. Reduction of WBC pathway activity causes fewer than three VPCs to adopt vulval fates, resulting in vulvaless (Vul), protruding vulva (Pvl), or egg laying defective (Egl) phenotypes (Eisenmann *et al.* 1998; Gleason *et al.* 2002, 2006). Conversely, overactivation of the WBC pathway causes more than three VPCs to adopt vulval fates, resulting in a multivulva (Muv) phenotype (Gleason *et al.* 2002; Gleason *et al.* 2006). Except for the Hox gene *lin-39*, no targets of the WBC pathway in VPC fate specification have been identified (Eisenmann *et al.* 1998)

The seam cells are lateral epithelial cells arranged in a single row running the length of the animal on both sides (Joshi *et al.* 2010; Chisholm and Hsiao 2012). Ten seam cells per side (H0–H2, V1–V6, and T) are born during embryogenesis but do not divide. Later, during each of the four larval stages (L1–L4), most seam cells divide asymmetrically in a stem cell–like manner to give rise to an anterior hypodermal daughter that differentiates and fuses with the surrounding skin and a posterior daughter that retains the seam cell fate and the ability to divide further (Joshi *et al.* 2010). During the second larval stage, six seam cells also divide symmetrically to generate two seam cell daughters, raising the number to 16 seam cells per side. After their final division in the fourth larval stage, the seam cells exit the cell cycle, differentiate, and fuse to form a long, single-cell syncytium that secretes a cuticular structure called the alae (Joshi *et al.* 2010). The asymmetric division of larval seam cells is regulated by the WBA pathway (Herman and Horvitz 1994; Whangbo *et al.* 2000; Herman 2001; Takeshita and Sawa 2005; Goldstein *et al.* 2006; Mizumoto and Sawa 2007; Huang *et al.* 2009c; Gleason and Eisenmann 2010; Ren and Zhang 2010; Banerjee *et al.* 2010). Reduction of the WBA pathway activity during larval life causes seam-fated daughters of a seam cell division to adopt hypodermal fates, resulting in fewer adult seam cells, whereas an increase in the WBA pathway activity causes hypodermal-fated daughters to adopt the seam cell fate, resulting in too many seam cells (Huang *et al.* 2009c; Gleason and Eisenmann 2010; Ren and Zhang 2010; Banerjee *et al.* 2010). We have recently shown that GATA factor–encoding genes *egl-18* and *elt-6* function as downstream targets of the WBA pathway required to retain the posterior seam cell daughter fate during these larval asymmetric seam cell divisions (Gorrepati *et al.* 2013).

Although the mechanisms of the two *C. elegans* β-catenin-dependent Wnt pathways differ, the common outcome is the regulation of target gene expression by a nuclear complex between a β-catenin (BAR-1 or SYS-1) and the sole *C. elegans* TCF homolog, POP-1. A key question is how formation of a β-catenin/POP-1 complex leads to distinct cellular responses in different tissues and cell types, such as the VPCs and seam cells. As a first step in addressing this question, the target genes activated by Wnt signaling in distinct processes must be identified. Previously, molecular genetic analyses have identified a few transcription factors functioning downstream of Wnt signaling in different *C. elegans* Wnt-mediated processes (Eisenmann *et al.* 1998; Jiang and Sternberg 1998; Maloof *et al.* 1999; Streit *et al.* 2002; Maduro *et al.* 2005; Shetty *et al.* 2005; Arata *et al.* 2006; Lam *et al.* 2006; Bertrand and Hobert 2009; Gorrepati *et al.* 2013). However, attempts to identify the broad range of Wnt signaling targets governing a particular process at a specific time in development using techniques such as microarray analysis or RNAseq have only been recently undertaken in *C. elegans*. Recently, our laboratory utilized global activation of Wnt signaling by a dominant activating BAR-1/β-catenin and microarray analysis to identify more than 100 potential Wnt pathway targets functioning in larval life, and revealed a previously unknown role for Wnt signaling in adult cuticle development (Jackson *et al.* 2014). Taking the opposite approach, van der Bent *et al.* (2014) used microarray analysis to identify more than 1000 genes differentially regulated between wild-type and *bar-1* null mutant strains. In addition to these genomic methods, a bioinformatic search utilizing an extended POP-1/TCF binding site also was successful at identifying novel Wnt responsive genes (Bhambhani *et al.* 2014). However, all of these methods analyzed target genes at the level of the whole animal and may have missed cell type–specific targets of Wnt signaling functioning in only a few cells, such as the VPCs (six cells) and seam cells (32 cells).

The control of cell fate specification in the VPCs and the asymmetric division of the seam cells by Wnt signaling made these cell types attractive candidates for attempting to identify cell type–specific Wnt targets in the worm. To achieve this, we used heat shock inducible dominant variants of BAR-1/β-catenin and POP-1/TCF to activate or inhibit Wnt signaling, respectively, at a specific time point in larval development (Gleason *et al.* 2002; Korswagen *et al.* 2002; Gleason and Eisenmann 2010; Jackson *et al.* 2014). To selectively enrich for mRNAs expressed in seam cells and VPCs, we utilized the "mRNA tagging method," which has been successfully used in combination with microarray analysis to catalog gene expression patterns from small numbers of cells (Roy *et al.* 2002; Kunitomo *et al.* 2005; Pauli *et al.* 2006; Von Stetina *et al.* 2007a,b; Takayama *et al.* 2010). This novel combination of differential activation of Wnt signaling with the "mRNA tagging method" and microarray analysis allowed us to identify 239 Wnt responsive genes differentially regulated by Wnt signaling in the seam cells or VPCs. Preliminary characterization of 50 known and novel genes was pursued to validate their expression in these cell types and to determine any possible role in their development or behavior. We found seven Wnt target genes that affect seam syncytium and alae formation both as a consequence of an effect on total seam cell number (*cki-1*, *cdk-4*, *kin-10*, *mlt-11*, *nhr-23*) and also independent of it (*bus-8*, *K10D6.2*). Two other Wnt responsive genes (*pak-1* and *lin-1*) were shown to function in VPC fate specification. This work validates the use of these different techniques in combination for the identification of cell type–specific signaling pathway targets *in vivo* in *C. elegans* and extends our previous work on the role of Wnt signaling in the regulation of seam cell and VPC fate specification by identifying new Wnt targets that control various aspects of the behavior of these cells during larval development.

## Materials and Methods

### Strains and alleles

Bristol N2 variety of *C. elegans* was used as the wild-type strain. All experiments were performed at 20° unless otherwise noted. The genes and alleles used in this work are described in Wormbase (Harris *et al.* 2010; Yook *et al.* 2012).

LGI: *kin-10*(*ok1751*); LGII: *rrf-3*(*pk1426*); LGIII: *unc-119*(*ed3*); LGIV*: dpy-20*(*e1282*), *unc-30*(*e191*); LGX: *pak-1*(*ok448*), *osm-11*(*rt142*)

Strains used:

*deIs10: dpy-20*(*e1282*); *unc-30*(*e191*); *Is*[*bar-1e*::*flag_3x_*::*pab-1; unc-30*(*+*); *ajm-1*::*gfp*]
*huIs1*: *dpy-20*(*e1282*); *unc-30*(*e191*); *Is*[*hsp-16.2*::*delNTbar-1*; *dpy-20(+)*] (Gleason *et al.* 2002)
*deIs26*: *dpy-20*(*e1282*); *unc-30*(*e191*); *Is*[*hsp-16.2*::*delNTpop-1*; *dpy-20(+)*]
*sIs12963*: [*F09D12.1p*::*gfp*]; *dpy-5*(*e907*) (McKay *et al.* 2003). This strain is referred to as *grd-10*::*gfp* in this work.
*scm*::*gfp: unc-119(e2498) III*; *wIs51[scmp*::*gfp*; *unc-119(+)]* (Gleason and Eisenmann 2010)

### Generation of strains for mRNA tagging

The *bar-1_e_* enhancer element fused to the minimal *pes-10* promoter was PCR amplified from the *VPC_e_*::*pes-10*::*GFP* vector (Natarajan *et al.* 2004) with primers containing *Kpn*I restriction sites. This 1131-bp PCR product was cloned upstream of Flag_3X_-PAB-1 in the pSV15 vector (Von Stetina *et al.* 2007a). The resulting cloned vector pBJ1000 (50 ng/µl) was coinjected with transformation markers *unc-30(+)* (100 ng/µl of pSC11) and *ajm-1*::*gfp* (50 ng/µl of pJS191) into *dpy-20*(*e1282*) *unc-30*(*e191*); *huIs1* worms using standard injection protocols (Mello and Fire 1995), and one line was selected for integration using gamma irradiation (3850 RADs). A transgenic line showing more than 95% transmission of the integrated array was backcrossed six times to generate *dpy-20(e1282) unc-30(e191)*; *deIs10 [VPCe*::*flag_3x_*::*pab-1;unc-30(+)*; *ajm-1*::*gfp]*. Transgenic worms bearing *hs*::*ΔNTpop-1* were generated by injecting pHCK28 (50 ng/µl) (Korswagen *et al.* 2000) and the coinjection marker *dpy-20*(+) (100 ng/µl of pMH86) into *dpy-20*(*e1362*) *unc-30*(*e191*) worms as above. Array integration into the genome was performed in the same manner as described above to generate *dpy-20*(*e1282*) *unc-30*(*e191*); *deIs26* [*hs*::*ΔNTpop-1*; *dpy-20*(*+*)]. The *deIs10* worms were crossed with *huIs1* and *deIs26* animals to generate the experimental strains *dpy-20*(*e1282*) *unc-30*(*e191*); *deIs10*; *huIs1* and *dpy-20*(*e1282*) *unc-30*(*e191*); *deIs10*; *deIs26*, respectively, which were used for mRNA tagging and microarray analysis.

### Heat shock and mRNA tagging protocol

Mixed-stage embryos from gravid adults of experimental strains (*deIs10*; *huIs1* and *deIs10*; *deIs26*) and control strains (N2 and *deIs10*; *scm*::*gfp*) were hatched overnight in 50 ml of M9. The synchronized L1s obtained were grown for 26 hr (L2/L3 molt) at 20°, heat shocked at 37° for 30 min, and then recovered for 1 hr at 20°. mRNA tagging was performed as previously described with a few modifications (Von Stetina *et al.* 2007b**)**. Briefly, heat shocked and recovered animals were treated with 20% paraformaldehyde for 1 hr at 4°, sonicated utilizing a Branson 450 Sonifier (2 W output, 20% duty cycle, 6 pulses for 6 rounds), homogenized for 5 min using a 7-ml Dounce homogenizer, and centrifuged at 11,750 rpm for 20 min at 4° to pellet worm debris; 1 ml of worm lysate supplemented with 10 μl rRNAsin (Promega #N-2515) and 8 μl 200 mM ribonucleoside vanadyl complex (Sigma #R-3380) was added to 100 μl prewashed anti-FLAG beads (Sigma #F-2426) and rocked at 4° for 12–16 hr. Beads were washed six times in low-salt homogenization buffer (25 mM NaCl, 20 mM HEPES, 1 mM EGTA, 1 mM EDTA, 0.6 mg/ml Heparin, and 10% glycerol) at 4° with rocking in between. Reversal of crosslinked FLAG::PAB-1/mRNA complexes was accomplished by adding 125 μl of elution buffer (50 mM Tris, 10 mM EDTA and 1.3% SDS) and 10 μl rRNAsin and rolling at 65° for 30 min; beads were spun down to obtain the eluted mRNA. After a second elution, the eluates were pooled and mRNA was extracted using the Trizol method.

### mRNA amplification and microarray analysis

Biological triplicates of mRNA samples obtained by mRNA tagging and coimmunoprecipitation from *deIs10*; *huIs1*, *deIs10*; *deIs26*, *deIs10;scm*::*gfp*, and N2 were subjected to DNase treatment. A total of 50 ng of mRNA was used as the starting sample for the RNA amplification process using the WT-Ovation Pico System (NuGEN #3300) to generate 3–6 μg of amplified cDNA. Fragmentation and biotin labeling of the amplified cDNA samples were performed out using the FLOvation cDNA Biotin Module V2 kit (NuGEN #4200). The labeled cDNA was hybridized to Affymetrix *C. elegans* GeneChip array (Affymetrix #900384**)** containing 22,500 probe sets targeting 21,150 unique *C. elegans* transcripts (Affymetrix 2004). The hybridization intensities obtained from each experiment were normalized using the Robust Multichip Average (RMA) analysis, and the detection calls and directional change calls were given based on Affymetrix’s MicroArray Suite 5 (MAS5) software analysis.

Putative Wnt pathway targets were selected based on the following criteria: (1) an average fold change of 1.5-fold or more in *deIs10*; *huIs1* (Wnt pathway overactivation) compared to control (N2); (2) present and increased calls in at least two of three biological replicates; (3) ANOVA *P* ≤0.05; and (4) ratio of average fold change ≥1.5-fold between Wnt pathway overactivated (*deIs10*; *huIs1*) and underactivated (*deIs10*; *deIs26*) conditions. These criteria identified 238 Wnt targets to which *kin-10* was added, although its *P* was more than 0.05 (*P* = 0.12), increasing the total list of identified targets to 239. The statistical significance of the overlap between our gene lists and those identified by others (Table 1) was calculated using a web-based hypergeometric distribution calculator designed by Jim Lund (University of Kentucky, Lexington, KY, USA) (http://nemates.org/MA/progs/overlap_stats.html).

**Table 1:** Conditional Wnt activation coupled with mRNA tagging enriches for cell type–specific transcripts and Wnt-regulated genes

Cell/Tissue Type	Reference	No. of Genes	No. of Common Genes	R-factor	Probability
Larval seam cells	Wormbase release 220	392	24	5.7	8.8×10^−12^
Larval and adult vulval cells	Wormbase release 220	305	8	2.4	0.02
Embryonic muscle	Fox *et al.* 2007	389	4	0.9	0.41
Embryonic/larval body wall muscle	Spencer *et al.* 2011	123	0	0	0.26
Embryonic/larval A class neurons	Spencer *et al.* 2011	108	0	0	0.31
Embryonic/larval pan-neural	Spencer *et al.* 2011	104	0	0.9	0.31
Embryonic/larval intestine	Spencer *et al.* 2011	298	0	0	0.04
Global Wnt targets	Jackson *et al.* 2014	110	24	20.1	9.6×10^−25^
*bar-1(ga80)* downregulated targets	van der Bent *et al.* 2014	710	25	3.3	2.3×10^−7^
*bar-1(ga80)* upregulated targets	van der Bent *et al.* 2014	425	10	2.2	0.01

Shown is a comparison of our list of 239 seam/VPC Wnt-regulated genes identified by conditional Wnt pathway activation and mRNA tagging to sets of genes known to be expressed in the seam cells, vulval cells, and other embryonic and larval tissues, and to the list of Wnt-regulated genes identified previously using these reagents without tissue-specific transcript enrichment (global Wnt targets). The statistical significance of the overlap between two lists was calculated based on a hypergeometric distribution. A representation (R) factor >1 indicates more overlap than expected between two independent groups of genes, whereas an R factor <1 indicates less overlap than expected (Lund *et al.* 2002). Genes identified in this work that are known to be expressed in the seam cells or vulval cells are indicated in Table S1. Genes in common between this work and that of Jackson *et al.* (2014) are indicated in Table S2. Genes in common between this work and that of van der Bent *et al.* (2014) are indicated in Table S3.

### qRT-PCR analysis

For qRT-PCR analysis, 1–1.5 μl cDNA (50 ng/μl) was mixed with 200 nM gene-specific forward and reverse primers and 12.5 μl of SYBR Green supermix in a final volume of 25 μl. PCR was performed on a BioRad iCycleriQ multicolor real-time PCR detection system (technical triplicates of each reaction were performed). The fold change of target genes was determined from the observed Ct (cycle threshold) values and calculated using the Pfaffl equation, (E target)^ΔCt target (control-treated)^**/**(E reference)^ΔCt ref (control-treated)^ (Pfaffl 2001), with a reference gene of *gpd-2*. A minimum of three biological replicates of control and experimental samples were analyzed.

### Transcriptional reporter construct

To create *promoter*::*yfp* transcriptional reporter constructs, the intergenic region between the ATG of the gene of interest and the next upstream gene was PCR amplified with gene-specific primers containing Gateway attachment sites and cloned into pBJ101, which contains a 2XNLS::YFP coding sequence and the *unc-119(+)* minigene (Jackson *et al.* 2014). Oligonucleotide sequences are provided in Supporting Information, Table S7. Each *promoter*::*yfp* construct (150 ng/µl) was coinjected with *ajm-1*::*gfp* (pJS191; 50 ng/µl) into *unc-119*(*ed3*) worms. Transgenic worms were detected based on the ability to move and expression of *ajm-1*::*gfp*. At least two independent lines were analyzed for each gene.

### RNA interference

Sequence-verified RNAi clones used in this work were obtained from the Ahringer library (Kamath and Ahringer 2003) and the Vidal library (Rual *et al.* 2004a) (gifts from Dr. Iqbal Hamza of UMCP and Dr. Mark Wilson of JHU). RNAi on L1 and P0 animals was performed as described (Kamat *et al.* 2001; Liu *et al.* 2014). Worms of the appropriate age were analyzed on a Zeiss Axioplan 2 equipped with Nikon DXM1200 digital camera for imaging.

### Immunostaining

Immunostaining of worms was performed as described (Duerr 2006). Anti-FLAG mouse monoclonal antibody M2 (Sigma #F3165) was used at 1/1000 for PAB-1 detection and MH27 mouse monoclonal antibody (Developmental Studies Hybridoma Bank) at 1/100 for detection of the junctional molecule AJM-1. Stained worms were mounted and were analyzed on a Zeiss Axioplan 2 equipped with a Nikon DXM1200 digital camera for imaging.

## Results and Discussion

### Conditional Wnt pathway activation, mRNA tagging, and microarray analysis identify 239 putative Wnt pathway target genes in the seam cells and VPCs

**Figure 1:**
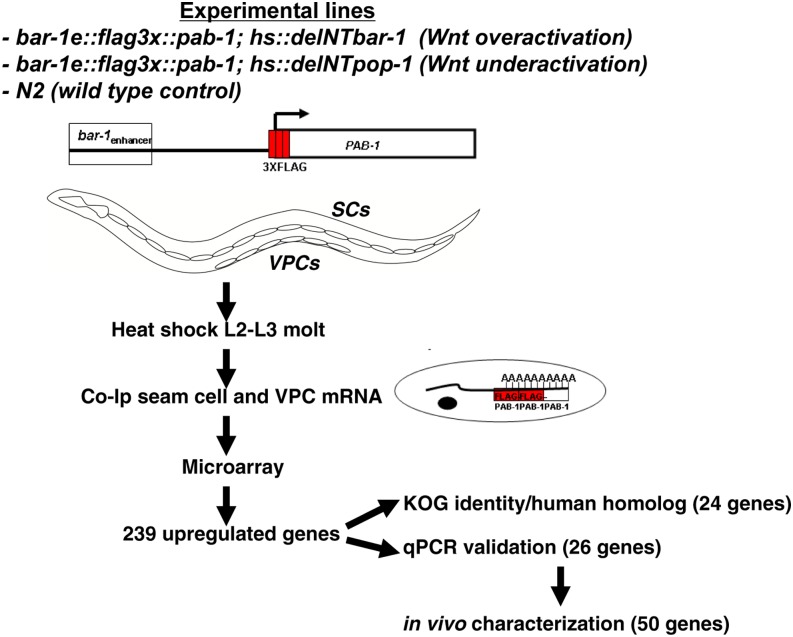
Conditional Wnt pathway activation, mRNA tagging, and microarray analysis to identify seam and VPC-specific Wnt targets. Outlined here is the procedure used to conditionally activate the Wnt pathway in L2/L3 worms by heat shock and enrich for transcripts expressed in the seam cells and VPCs using the mRNA tagging method. An SC+VPC-specific enhancer element from the *bar-1* gene (*bar-1_e_*) was used to drive FLAG-tagged poly-A tail binding protein (PAB-1) in the seam cells and VPCs. Crosslinked FLAG-PAB-1-mRNA complexes were immunoprecipitated, enriching for transcripts from those cell types. Transcript profiles after heat shock from the three indicated strains were determined using a *C. elegans* genomic DNA microarray, and transcripts upregulated in response to Wnt pathway activation were identified. A subset of genes was subject to further *in vivo* characterization based on criteria of their identity or independent qPCR validation.

To identify genes regulated by Wnt signaling in the larval seam and vulval precursor cells, three methods were used: (1) conditional Wnt pathway activation or inhibition at a defined time during larval development; (2) mRNA tagging to enrich for seam and VPC transcripts; and (3) microarray analysis to identify differentially regulated genes (Figure 1). We conditionally activated Wnt signaling at the time of the L2/L3 molt using *hs*::*delNTbar-1*, which encodes a heat shock inducible variant of BAR-1/β-catenin lacking the amino terminal region required for degradation in the absence of signaling (Gleason and Eisenmann 2010; Jackson *et al.* 2014). Expression of this truncated variant at the L2/L3 molt causes an activated Wnt pathway phenotype in both the VPCs (Gleason *et al.* 2002) and seam cells (Gleason and Eisenmann 2010), and we previously used this reagent to identify Wnt signaling target genes by microarray analysis (Jackson *et al.* 2014). We conditionally inhibited Wnt signaling via expression of a heat shock inducible variant of POP-1/TCF that lacks the N-terminal β-catenin binding domain required for activation of target gene expression (*hs*::*delNTpop-1*). Expression of this dominant negative ΔNTPOP-1 has been shown to cause phenotypes consistent with reduced Wnt signaling in both neurons and seam cells during larval development (Korswagen *et al.* 2000; Gleason and Eisenmann 2010), and to reduce expression of Wnt target genes (Jackson *et al.* 2014). We activated or inhibited Wnt signaling by growing experimental (*hs*::*delNTbar-1*, *hs*::*delNTpop-1*) and control strains in triplicate to the L2/L3 molt, subjecting them to a single heat shock for 30 min at 37°, then collecting mRNA for analysis 1 hr after heat shock (Jackson *et al.* 2014).

In our previous analysis of the transcriptional response to activated Wnt signaling in larvae, we characterized gene expression from the entire animal (Jackson *et al.* 2014). To enrich for transcripts from the small number of larval cells of interest to us, the seam cells (32 cells) and VPCs (6 cells), we used the "mRNA tagging" method (Roy *et al.* 2002). In this technique, a FLAG tagged version of the polyA binding protein PAB-1 is expressed using a cell type–specific enhancer and mRNAs are crosslinked to FLAG-PAB-1 *in vivo* and coimmunoprecipitated *in vitro* (Figure 1). The mRNA tagging method has been successfully used to generate inventories of genes expressed in various *C. elegans* tissues (Roy *et al.* 2002; Kunitomo *et al.* 2005; Pauli *et al.* 2006; Von Stetina *et al.* 2007a; Von Stetina *et al.* 2007b; Takayama *et al.* 2010). FLAG-PAB-1 expression in our experiments was under the control of an enhancer from the *bar-1* gene that is active in larval seam cells and VPCs (*bar-1_e_*) (Natarajan *et al.* 2004) (Figure S1). mRNA bound to FLAG-PAB-1 was coimmunoprecipitated from Wnt activated and Wnt inhibited strains, and these seam and VPC enriched transcript pools were used for microarray analysis on Affymetrix *C. elegans* genome arrays as previously described (Jackson *et al.* 2014). Gene expression from the experimental strains was compared to that in control strains. After the application of several selection criteria (see *Materials and Methods*) we identified 239 genes upregulated in response to overexpression of the dominant BAR-1 protein, corresponding to ∼1% of the unique *C. elegans* transcripts represented on the genome array (Table S1). We refer to these genes as larval "Wnt pathway-regulated" or "Wnt target" genes.

If these genes are potential Wnt targets from the seam and vulval cells, we would predict overlap with genes known to be expressed or function in the larval seam cells or VPCs, and known or putative Wnt pathway target genes. As shown in Table 1, genes expressed in the larval seam cells were over-represented in our list of cell type–specific Wnt pathway–regulated genes (R-factor = 5.7; *P* = 8.8×10^−12^). Genes expressed in the vulval cells were also over-represented (*P* = 0.02), whereas genes expressed in embryonic and larval body wall muscle cells, neurons, and intestinal cells (Spencer *et al.* 2011) were not (R factor <1 in each case; Table 1). We also compared our list of genes with those Wnt-regulated genes identified previously using these reagents (Jackson *et al.* 2014). Twenty-four of the 110 genes activated by Wnt signaling in that study were present among the Wnt pathway–regulated genes identified here (R factor = 20.1; *P* = 9.6×10^−25^) (Table 1, Table S2). Recently, microarray analysis was also used to identify more than 1100 genes showing altered expression at the L4 stage in a strain carrying a loss-of-function mutation in the gene encoding the beta-catenin BAR-1 (van der Bent *et al.* 2014). We found 35 genes in common between these *bar-1*-regulated genes and our 239 genes; 25 of the genes were downregulated upon loss of BAR-1 (van der Bent *et al.* 2014) and upregulated upon expression of activated BAR-1 (this work), a significant overlap (Table 1, Table S3). Thirteen of these genes (*col-138*, *col-38*, *col-49*, *col-71*, *C04F1.1*, *C18A11.4*, *dao-4*, *dpy-11*, *F25E5.2*, *grd-2*, *hog-1*, *R07B1.5*, and *T07G12.3*) were downregulated at the L4 stage upon loss of *bar-1* (van der Bent *et al.* 2014) and upregulated in both our previous work using overexpression of BAR-1 at the L2/L3 molt (Jackson *et al.* 2014) and in this work (Table S3); therefore, they are strong candidates for larval targets of the WBC pathway. Finally, of the small number of previously known direct Wnt pathway targets, our mRNA tagging approach identified the Hox gene *mab-5*, which is a predicted target of the WBC pathway in the seam cell V5.p (Hunter *et al.* 1999; Maloof *et al.* 1999) . Hox gene *lin-39*, a predicted target of the WBC pathway in the VPCs (Eisenmann *et al.* 1998; Hoier *et al.* 2000; Wagmaister *et al.* 2006), was upregulated 1.5-fold upon Wnt pathway overactivation, but failed to meet the target selection criterion of *P* ≤ 0.05. The other known Wnt signaling targets are expressed in other cell types or developmental stages, and are not predicted to be identified by our method. Taken together, these results suggest that combining conditional activation of the Wnt signaling pathway with mRNA tagging and microarray analysis was successful in identifying putative Wnt pathway target genes with a high representation of genes expressed in the seam cells and vulval cells.

### Characterization of Wnt pathway-regulated genes

Analysis of the 239 Wnt pathway-regulated genes by DAVID (Huang *et al.* 2009a,b) identified 57 genes in four significantly enriched, functionally related groups: structural constituents of cuticle; transcription factors; signaling proteins; and protein kinases (Table S4). Thirteen of the 57 genes (∼24%) are expressed in the seam cells and/or the developing vulva (Yook *et al.* 2012) (Table S4). The largest class of enriched genes includes those encoding components of the worm’s outer surface, the cuticle, which is synthesized by hypodermal cells (Page and Johnstone 2007). In our previous analysis of Wnt-activated genes in the larva, we identified a subset of cuticle collagen genes that are normally expressed in the mid L4 stage, but that can be expressed earlier in response to ectopic Wnt signaling (Jackson *et al.* 2014). Reduction of function for these genes caused a defect in adult cuticle integrity (Jackson *et al.* 2014). The identification of a large number of *col* genes in the current assay confirms the result from the previous analysis and supports the hypothesis of a previously unknown role for Wnt signaling in adult cuticle synthesis (Jackson *et al.* 2014; van der Bent *et al.* 2014). We did not pursue further characterization of the cuticle component genes identified in this list of seam and VPC Wnt target genes.

To identify new Wnt pathway–regulated genes for further analysis, we took two approaches. First, we categorized the putative Wnt pathway target genes based on their KOG identity (EuKaryotic Orthologous Groups) (Yook *et al.* 2012) and occurrence of a known human ortholog (Shaye and Greenwald 2011). This method identified 124 genes that encode transcription factors, signaling molecules, kinases, enzymes, collagens, or other proteins. From these broad categories we selected 24 genes to examine further; nine were chosen because they had human homologs, and the others were selected in such a way to span a range of Wnt activation levels (they ranged from 1.5× to 23.5× increased expression). Second, we repeated the mRNA tagging experiment and performed qPCR to analyze gene expression levels for more than 100 of the putative target genes identified by microarray analysis (data not shown). Twenty-seven of the genes were upregulated in response to Wnt pathway activation when assayed by this method, and 12 of these were also downregulated in response to Wnt pathway inhibition (Table S5). We do not have an explanation for the low validation rate by qPCR; however, a similarly low validation rate was seen in our previous microarray analysis using these conditional activation/inhibition reagents (Jackson *et al.* 2014). Both of these studies used a low cutoff for inclusion in the data set, and we noted that the qPCR validation rate declines as one moves down the genes in rank order (L. Gorrepati, B. Jackson and D. Eisenmann, unpublished observations). Among the genes regulated by both activation and inhibition of Wnt signaling was the Wnt pathway negative regulatory component *pry*-1*/Axin*, which we previously showed is a Wnt-responsive target gene in *C. elegans* as in other species (Jackson *et al.* 2014). We chose 26 qPCR validated genes, including 17 genes that had no previous characterization, for further study. In this way we selected 50 seam cell/VPC Wnt pathway–regulated genes encoding previously known and novel gene products for further characterization (Table 2). In addition to these genes, we also identified the adjacent genes *egl-18* and *elt-6*, which encode GATA transcription factors previously shown to be required for seam cell fate specification in the embryo (Koh and Rothman 2001). In a separate analysis (Gorrepati *et al.* 2013), we showed that *egl-18* and *elt-6* function downstream of Wnt signaling in the larva seam cells to maintain the progenitor cell fate during larval seam cell divisions (these genes are not described further here).

**Table 2:** Wnt-regulated genes from the seam cells and VPCs selected for further characterization

Gene Function	Gene	Encodes	Fold Change Wnt Overactivation	Expression	Reference
Signaling	*che-14*	Dispatched homolog	4.1	Seam cells, vulva	Michaux *et al.* 2000
					Jarriault *et al.* 2008
	*grd-3*	Hedgehog-like protein	2.5	Seam cells	Aspock *et al.* 1999
					Mallo *et al.* 2002
	*grd-14*	Hedgehog-like protein	2.4	Seam cells	Hao *et al.* 2006
					McKay *et al.* 2003
	*hog-1*	Hedgehog-like protein	3.1	Other	Hao *et al.* 2006
	*old-2*	Protein tyrosine kinase	6.7	N.D.	
	*osm-11*	DOS domain-containing ligand	1.6	Seam cells, VPCs	Komatsu *et al.* 2008
					Singh *et al.* 2011
	*rab-8*	Ras GTPAse	2.1	Other	Meissner *et al.* 2011
	*rhgf-2*	Guanine nucleotide exchange factor	2.1	Other	Ruvinsky *et al.* 2007
					McKay *et al.* 2003
					Lin *et al.* 2012
Transcription regulation	*ldb-1*	LIM domain-binding factor	1.9	Vulva, other	Dupuy *et al.* 2007
					Cassata *et al.* 2000
					Wenick and Hobert 2004
					Reece-Hoyes *et al.* 2007
					Levin *et al.* 2012
	*lin-1*	Ets-domain-containing transcription factor	1.9	Other	Dupuy *et al.* 2007
					McKay *et al.* 2003
					Murray *et al.* 2012
	*mab-5*	Homeodomain transcription factor	3.1	P cells, other	Salser and Kenyon 1992
	*nhr-23*	Nuclear hormone receptor	2.7	Seam cells, hyp	Kostrouchova *et al.* 1998
					Feng *et al.* 2012
	*nhr-74*	Nuclear hormone receptor	4.8	Seam cells	Miyabayashi *et al.* 1999
	*nhr-113*	Nuclear hormone receptor	4.3	Seam cells	Reece-Hoyes *et al.* 2007
	*tbx-2*	T-box transcription factor	1.9	Other	Miyahara *et al.* 2004
					Roy Chowdhuri *et al.* 2006
					Smith and Mango 2007
Enzymes	*cdk-4*	Cyclin-dependent kinase	3.9	Seam cells, VPCs, other	Park and Krause 1999
	*dhs-5*	Dehydrogenase	2.8	Hyp, other	Wang *et al.* 2006
	*pak-1*	p21-activated kinase	1.6	Seam cell T, VPCs, other	Chen *et al.* 1996
					Lino and Yamamoto 1998
					Goh *et al.* 2012
	*glna-2*	Glutaminase	2.6	Hyp, other	McKay *et al.* 2003
	*F26E4.5*	Tyrosine kinase	3.5	N.D.	
	*bus-8*	Glycosyltransferase	2.9	Seam cells	Partridge *et al.* 2008
	*kin-10*	Casein kinase beta regulatory subunit	1.5	Other	Hu *et al.* 2006
					Meissner *et al.* 2011
Unknown	*nlp-25*	neuropeptide-like protein	10.0	Other	This work
	*T26E4.4*	Human FUT2 homolog	13.3	P cells, hyp	Jackson *et al.* 2014
	*C54C8.2*	Uncharacterized protein	3.1	Seam cells	Reece-Hoyes *et al.* 2007
Other	*cki-1*	Cell cycle inhibitor	3.8	Seam cells, VPCs, other	Hong *et al.*1998
					Feng *et al.* 1999
					Saito *et al.* 2004
					Fukuyama *et al.* 2003
					Fujita *et al.* 2007
	*col-49*	Collagen	23.5	Seam cells, P cells, hyp	Jackson *et al.* 2014
	*pry-1*	Scaffolding protein	4.2	Seam cells, VPCs, other	Korswagen *et al.* 2002
					Mizumoto and Sawa 2007
	*rsp-4*	RNA processing protein	1.7	Other	Kawano *et al.* 2000
	*sqv-7*	Nucleotide sugar transporter	2.2	Seam cells, VPCs	Hwang and Horvitz 2002
					McKay *et al.* 2003
					Dupuy *et al.* 2007
	*twk-5*	K channel	2.8	No expression	This work
	*add-1*	Cytoskeletal protein	3.7	Seam cells, hyp, other	McKay *et al.* 2003
					Dupuy *et al.* 2007
					Vukojevic *et al.* 2012
	*K10D6.2*	Membrane protein	2.7	Seam cells, other	Kunitomo *et al.* 2005
	*rhy-1*	Regulator of hypoxia-inducible factor	3.6	Vulva, hyp	Shen *et al.* 2006
	*Y71H2AL.1*	Calcium-binding protein	1.7	Other	Wagner *et al.* 2011
	*mlt-11*	Serine protease inhibitor	1.8	Seam cells, hyp	Frand *et al.* 2005
Unknown	*C28H8.1*	Human gene BCL7A ortholog	3	N.D.	
	*nspe-1*	Nematode-specific peptide	7.5	Other	This work
	*pepm-1*	PEPtidase M1 domain-containing protein	2.3	Vulva	Inoue *et al.* 2002
	*ttr-15*	TransThyretin-related protein	2	No expression	This work
	*ttr-3*	TransThyretin-related protein	3.6	P cells, hyp	This work
	*ttr-4*	TransThyretin-related protein	3.6	No expression	This work
	*B0454.5*	Uncharacterized protein	3.8	P cells, hyp	This work
	*C08E3.13*	Uncharacterized protein	2.2	vulva, other	Mochii *et al.* 1999
	*C14C11.7*	Uncharacterized protein	4.5	No expression	This work
	*K10D6.3*	Uncharacterized protein	5.2	No expression	This work
	*M04C9.1*	Uncharacterized protein	5.4	No expression	This work
	*Y37E11B.7*	Uncharacterized protein	7.7	No expression	This work
	*Y43C5A.3*	Uncharacterized protein	10.0	P cells, hyp	This work
	*Y55F3AM.10*	Uncharacterized protein	3.4	No expression	This work

Shown here are 50 seam/VPC Wnt pathway-regulated genes selected for further characterization grouped into broad classes based on the identity of the protein encoded by each gene. The fold change in expression in Wnt pathway overactivation condition observed by microarray analysis is shown. Also shown is each gene’s expression pattern (previously known or reported here). hyp indicates hypodermal expression not in the seam or vulva lineages (P cells, VPCs, vulva); other indicates known expression outside of hypodermal lineages

### Expression analysis of putative seam and VPC Wnt target genes

**Figure 2:**
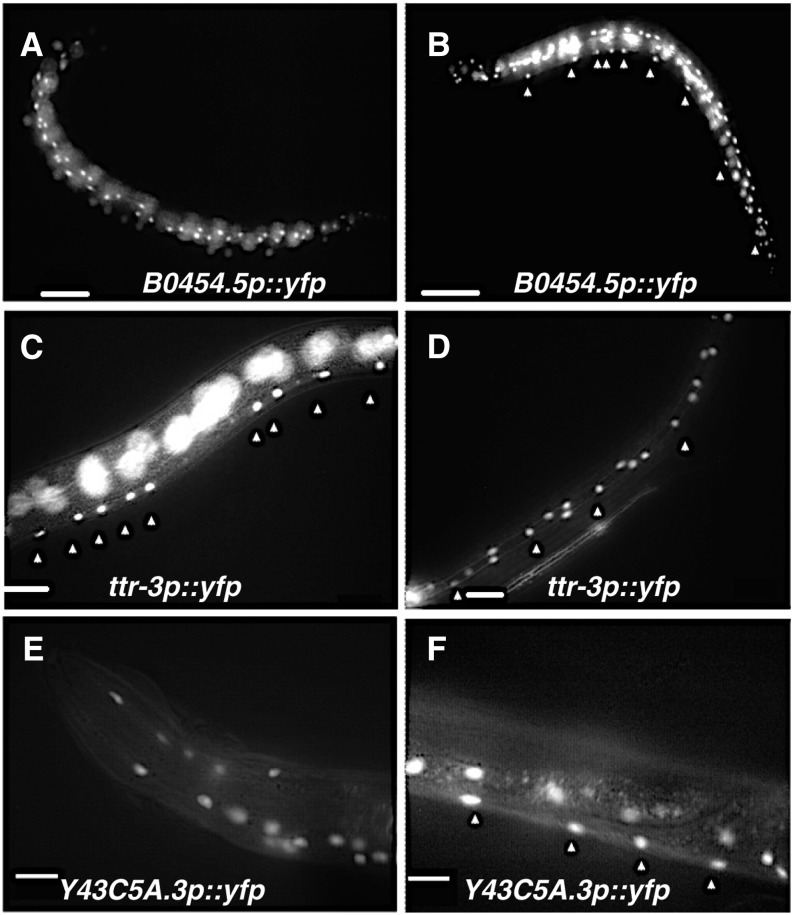
Transcriptional reporter analysis reveals putative Wnt targets are expressed in seam cells, hypodermis, and P cell descendents. Shown here are *promoter*::*YFP* expressing worms. (A and B) The target gene *B0454.5* is expressed in hypodermal cells (unlabeled) and P cell descendents (arrowheads in B) throughout larval life and in adults. (C and D) *ttr-3*::*YFP* is also expressed in the P cell descendents (arrowheads in C), seam cells (arrowheads in D), and hypodermal cells (unlabeled). (E and F) Expression of *Y43C5A.3*::*YFP* is shown in the hypodermal cells (E) and P cells (arrowheads in F). In (A) through (C), the out-of-focus fluorescence is from the seam and hypodermal cells, present in a different focal plane. Scale bars represent 50 μm in all figures.

Spatial expression data is known for 32 of the 50 putative Wnt target genes: 10 are expressed in seam cells, 3 are expressed in P cells (precursors to VPCs) and/or hypodermis, seven are expressed in seam and vulva, three are expressed in the vulva, and one is expressed in vulva and hypodermis; (Yook *et al.* 2012) (Table 2). Eight genes have known expression in other tissues but not in the seam and vulva (Yook *et al.* 2012) (Table 2); however, four of them have vulval or seam phenotypes when their function is compromised (*lin-1*, *kin-10*, *rsp-4*, and *hog-1*) (Ferguson and Horvitz 1985; Longman *et al.* 2001; Kamath and Ahringer 2003; Rual *et al.* 2004; Zugasti *et al.* 2005), suggesting they may be expressed in those cell types. Of the 18 genes with no known expression data, we generated 14 *promoter*::*yfp* constructs in which the upstream region from the start codon of the gene of interest to the next upstream gene was fused to 2X::NLS::YFP coding sequences (this work; Jackson *et al.* 2014). Five of 14 genes (*B0454.5*, *ttr-3*, *Y43C5A.3*, *col-49*, *T26E4.5*) were expressed in the hypodermis, seam cells, or P cell descendents, whereas two genes, *nlp-25* and *nspe-1*, showed expression in other tissues (Figure 2, Table 2) (Jackson *et al.* 2014). We failed to detect YFP expression for eight genes, suggesting the *promoter*::*yfp* constructs may not have included all possible regulatory elements (six of the eight genes were previously uncharacterized genes encoding novel proteins). In summary, of the 46 candidate genes examined, 63% showed expression in the seam or vulval lineages. Therefore, given their expression in the cell types of interest, and their upregulation in response to Wnt signaling, we consider these genes likely to be *bona fide* Wnt target genes in the seam cells and the vulval cells.

### Wnt targets genes function in seam cell and vulval development

The seam cells are lateral hypodermal cells extending in a single row from nose to tail that divide in a stem cell–like manner during each of the four larval stages to generate one daughter that retains the seam cell fate and the ability to divide further, and another daughter that differentiates and joins the syncytial skin of the growing worm (Joshi *et al.* 2010). The asymmetric division of the seam cells is known to be regulated by Wnt signaling (Gleason and Eisenmann 2010). After their final division in the L4 stage, the 16 seam cells on each side of the animal exit the cell cycle, terminally differentiate, and fuse with each other to form a single syncytial cell (Joshi *et al.* 2010). The differentiated seam cells secrete the specialized cuticular structure called alae (Joshi *et al.* 2010), which are visible as lateral parallel ridges running the length of the body in adult worms.

**Figure 3:**
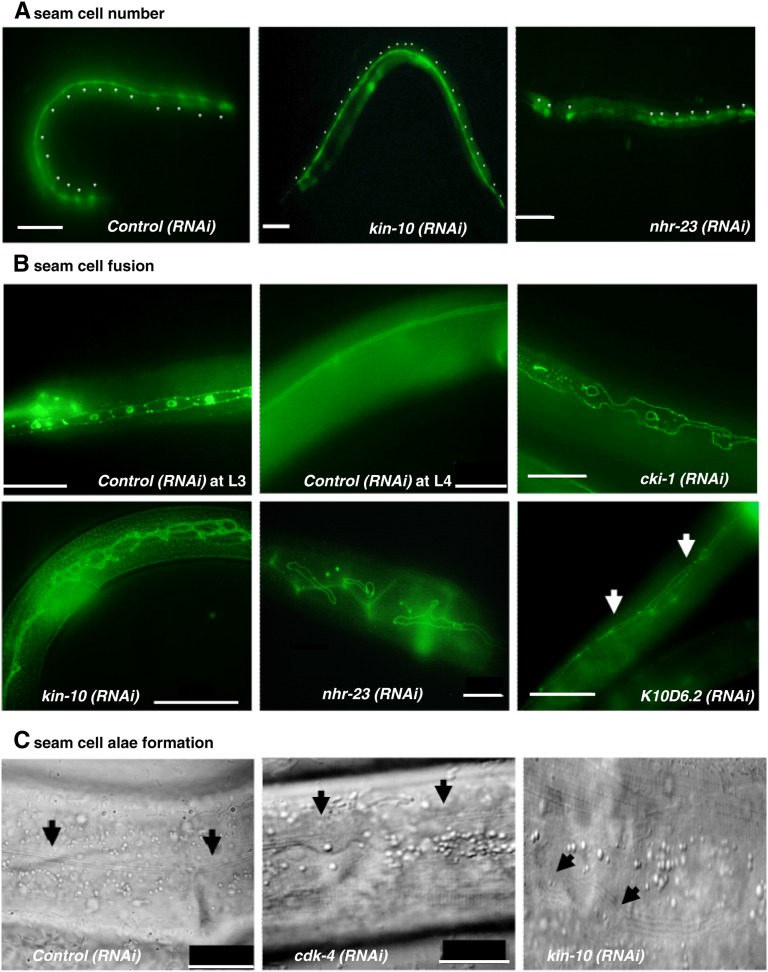
Putative Wnt targets influence seam cell number, formation of seam syncytium, and cuticular alae. (A) Seam cells (asterisks) in RNAi control, *kin-19(RNAi)*, and *nhr-23(RNAi)* young adult animals. All animals contain *grd-10*::*gfp*, which expresses GFP in the nucleus and cytoplasm of seam cells at all stages. RNAi control animals have 16 seam cells per side, whereas *kin-10(RNAi)* animals have additional seam cells and *nhr-23(RNAi)* animals have fewer seam cells. (B) After their last division in the L4 stage, seam cells fuse to form a single-cell syncytium that runs the length of the animal, which can be visualized by antibody staining for the junctional protein AJM-1. The first two panels show seam cells in RNAi control animals before (L3) and after homotypic fusion (late L4). At this later time, *cki-1(RNAi)* and *kin-10 (RNAi)* animals show ring-like malformations in the syncytium, *nhr-23(RNAi)* worms have gaps in the syncytium, and *K10D6.2(RNAi*) worms have gaps as well as unfused seam cells (arrow heads). (C) After their last division in the L4 stage, seam cells secrete a specialized cuticular structure called alae that is visualized as three to four parallel ridges running the length of the animal. The first panel shows a portion of the midbody cuticle of an RNAi control animal with intact alae. *cdk-4(RNAi)* animals show partial or incomplete alae (alae end at arrowheads with gap between), whereas *kin-10(RNAi)* worms exhibited misformed alae (arrow heads). Scale bars represent 50 μm in all figures.

We examined the role of 50 putative Wnt target genes in larval seam cell development using RNAi interference (RNAi). To increase the likelihood of observing novel phenotypes, we used an *rrf-3*(*pk1426*) background, which is hypersensitive to RNA interference (Simmer *et al.* 2002). We monitored seam cell numbers in RNAi-treated animals using the transcriptional reporter *grd-10p*::*gfp*, which expresses GFP in the nucleus and cytoplasm of all seam cells from early larval stages to adult **(**Figure 3, Table 3) (McKay *et al.* 2003). Alterations in seam cell number could indicate a defect in adoption or maintenance of the seam cell fate, execution of the asymmetric seam cell divisions, or seam cell survival (Gleason and Eisenmann 2010; Gorrepati *et al.* 2013). We also examined RNAi-treated animals for defects in seam cell fusion by antibody staining for the adherens junction protein AJM-1, and for defects in adult alae formation (Figure 3, Table 4, Table 5) (Page and Johnstone 2007). Defects in these properties could indicate a defect in the specification, survival, or terminal differentiation of seam cells.

**Table 3:** Reduction of function of five Wnt-regulated genes alters terminal seam cell number

Strain	n	Average Adult Seam Cell No.	Range
*control(RNAi)*	246	15.6	13–16
*cdk-4(RNAi)*	35	11.9*^a^*	7–16
*cki-1(RNAi)*	38	20.6*^a^*	17–26
*kin-10(RNAi)*	42	23.9*^a^*	16–36
*mlt-11(RNAi)*	43	10.5*^a^*	7–13
*nhr-23(RNAi)*	53	13.3*^a^*	9–16

All animals contain *rrf-3(pk1426)* and seam cell–specific marker *grd-10*::*gfp*; GFP+ cells were counted in young adult worms. The number of adult seam cells per side in control RNAi (feeding vector alone) and animals treated with RNAi against the indicated Wnt regulated genes is shown.

a *P* ≤ 0.001 (unpaired t-test) compared to control (RNAi).

**Table 4:** Wnt pathway components and Wnt-regulated genes influence seam syncytium formation

Strain	n	Normal (%)	Gaps (%)	Unfused (%)	Rings (%)
*control(RNAi)*	74	96	4	0	0
*bus-8(RNAi)*	72	64	26*^b^*	8*^b^*	1
*cdk-4(RNAi)*	36	53	47*^b^*	0	0
*cki-1(RNAi)*	33	12	85*^b^*	0	3
*K10D6.2(RNAi)*	56	0	70*^b^*	27*^b^*	3*^b^*
*kin-10(ok1751)*	51	39	2	0	59*^b^*
*kin-10(RNAi)*	51	16	0	0	84*^b^*
*mlt-11(RNAi)*	41	10	76*^b^*	2	12*^b^*
*nhr-23(RNAi)*	30	3	77*^b^*	17*^b^*	3*^a^*

Antibody staining against AJM-1 protein was used to visualize the seam syncytium in *rrf-3(pk1426)* animals treated with RNAi against the indicated Wnt-regulated genes. Normal indicates fused seam cells appearing as parallel tracks running the entire length of the animal; partial indicates gaps; unfused indicates presence of unfused seam cells; and rings indicates ring-shaped misformations along the seam syncytium. The percentage of animals with the indicated seam syncytium defects is shown.

a *P* ≤ 0.05.

b *P* ≤ 0.001 (Fisher’s exact test) compared to control (feeding vector alone).

**Table 5:** Wnt pathway components and Wnt-regulated genes influence adult alae formation

Strain	n	Normal (%)	Partial (%)	Absent (%)	Rings (%)
*control(RNAi)*	40	100	0	0	0
*bus-8(RNAi)*	37	8	30*^b^*	62*^b^*	0
*cdk-4(RNAi)*	31	36	45*^b^*	19*^b^*	0
*cki-1(RNAi)*	26	26	70*^b^*	4	0
*K10D6.2(RNAi)*	58	38	58*^b^*	7*^a^*	0
*kin-10(ok1751)*	52	0	10*^b^*	6*^b^*	84*^b^*
*kin-10(RNAi)*	31	6	7*^b^*	0	87*^b^*
*mlt-11(RNAi)*	41	2	20*^b^*	78*^b^*	0
*nhr-23(RNAi)*	34	0	24*^b^*	76*^b^*	0

*rrf-3(pk1426)* animals were treated with RNAi against the indicated Wnt-regulated genes and examined for defective adult alae formation. The percentage of worms with the indicated types of alae defects is shown. Normal indicates three parallel ridges running along the length of the animal; partial indicates gaps; absent indicates lack of alae; and rings indicates ring-like misformations along the length of the alae.

a *P* ≤ 0.05.

b *P* ≤ 0.001 (Fisher’s exact test) compared to control (feeding vector alone).

**Figure 4:**
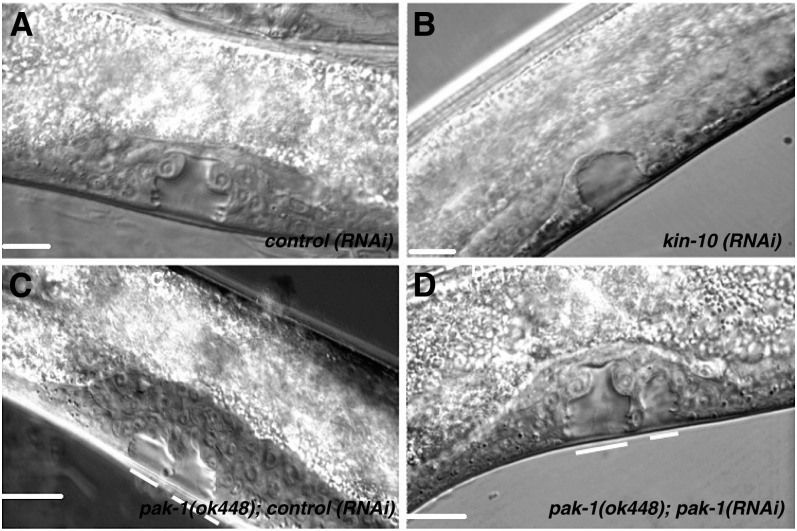
Reduction-of-function of putative Wnt targets affects L4 vulva formation. (A) In the control, three VPCs adopting primary and secondary fates in the L3 stage divide further and undergo morphogenesis, resulting in a Christmas tree–shaped L4 vulva. (B) When fewer than three VPCs adopt vulval fates, an underinduced phenotype can be seen as observed in *kin-10(RNAi)* worms. (C and D) The polarity of P7.p is reversed in Wnt signaling mutants resulting in a Bivulva phenotype. A similar phenotype was observed in *pak-1(RNAi)* worms and in *pak-1(ok448)* mutants. Scale bars represent 50 μm.

We also tested the involvement of the putative Wnt target genes in vulval precursor cell (VPC) fate specification by observing L4 vulval formation in RNAi-treated animals (Figure 4, Table 6). Wnt signaling is known to function in the specification or maintenance of the VPC fate, as well as in the asymmetric division of the VPCs P5.p and P7.p (Eisenmann *et al.* 1998; Eisenmann and Kim 2000; Gleason *et al.* 2002, 2006; Green *et al.* 2008). Abnormalities in vulval morphology observed at the L4 stage could indicate a defect in VPC fate specification or fate execution, vulval cell formation, or vulval morphogenesis (Eisenmann and Kim 2000).

**Table 6:** Wnt-regulated genes required for proper L4 vulva formation

Strain	n	Normal (%)	Underinduced (%)	Overinduced (%)	Bivulva (%)	Other (%)
*control (RNAi)*	81	98	0	0	0	2
*lin-1(RNAi)*	35	6	0	91*^b^*	0	3
*kin-10(RNAi)*	45	4	85*^b^*	4*^b^*	0	7*^b^*
*pak-1(ok448)*	61	80	5	0	13*^b^*	2
*pak-1(RNAi)*	63	90	2	0	8*^a^*	0

The top shows the percentage of *rrf-3(pk1426)* animals showing L4 vulval defects upon RNAi treatment for *lin-1* and *kin-10*. Normal indicates the characteristic Christmas tree–shaped L4 vulva formed when three VPCs adopt primary and secondary fates and divide further. Underinduced indicates improper L4 vulva formed when fewer than three VPCs adopt vulval fates. Overinduced indicates multiple vulval invaginations that form when more than three VPCs adopt vulval fates. Bivulva indicates a reversal in the polarity of the VPC P7.p resulting in an extra invagination.

a *P* ≤ 0.05.

b *P* ≤ 0.001 (Fisher’s exact test) compared to control (RNAi).

Of the 50 genes examined, five genes (*cdk-4*, *cki-1*, *kin-10*, *mlt-11*, and *nhr-23*) showed alterations in adult seam cell number (Table 3), seven genes (*bus-8*, *cdk-4*, *cki-1*, *kin-10*, *mlt-11*, *nhr-23*, and *K10D6.2*) showed defects in seam syncytium and alae formation (Table 4, Table 5), and three genes (*lin-1*, *kin-10*, and *pak-1*) had defects in L4 vulva formation (Table 6). We briefly describe these nine genes below.

### Wnt target genes that affect seam cell fate specification and terminal differentiation

#### cdk-4 (cyclin-dependent kinase 4):

CDK-4 forms a complex with cyclin D1 (CYD-1 in *C. elegans*) that regulates progress from the G1 to S phase of the cell cycle (van den Heuvel 2005). In *C. elegans*, CDK-4 is expressed during larval life in many cells, including the Pn.p cells, VPCs, and seam cells, and *cdk-4* RNAi-treated animals have defects in cell cycle progression for many postembryonic lineages (Park and Krause 1999). As expected for a positive cell cycle regulator, we found that *cdk-4(RNAi)* animals had fewer seam cells as adults (11.9 SCs/side, compared to an average of 15.6 in control RNAi animals) (Table 3); 47% of *cdk-4(RNAi)* worms displayed gaps in the seam syncytium (Table 4), whereas 64% of treated animals had missing or abnormal cuticular alae (Table 5, Figure 3K). These latter phenotypes are most likely due to the decrease in seam cell number observed in these animals, leading to gaps in the linear array of seam cells along the body axis. In humans, the cyclin D1 gene is a transcriptional target of Wnt signaling (Shtutman *et al.* 1999; Tetsu and McCormick 1999); however, the worm *cyd-1* gene did not pass our criteria for selection of Wnt pathway targets. These results suggest that *C. elegans* may use a different mechanism to activate cell proliferation in response to Wnt signaling, perhaps by upregulation of expression of *cdk-4* rather than its cyclin partner.

#### cki-1 (cyclin-dependent kinase inhibitor 1):

Curiously, the *cki-1* gene was also found as a potential Wnt target gene in our analysis. The cyclin-dependent kinase inhibitor CKI-1 is homologous to the mammalian Cip/Kip family of CKI proteins, which inhibit CDKs functioning in the G1 to S phase transition (Clayton *et al.* 2008). Loss of function of *cki-1* results in extra cell divisions in the embryo as well as in many postembryonic cell lineages, including the VPCs and seam cells (Hong *et al.* 1998; Fukuyama *et al.* 2003; Nimmo *et al.* 2005; Kagoshima *et al.* 2007; Clayton *et al.* 2008; Buck *et al.* 2009). As expected, we observed an increased seam cell number in *cki-1(RNAi)*-treated adults (20.6 SCs/side) (Table 3). Curiously, although *cki-1(RNAi)* worms had more seam cells, more than 85% of animals had gaps in the seam syncytium or showed misformed syncytium (Table 4, Figure 3F). In addition, the adult alae were missing or only partially formed in 74% of adult worms (Table 5). These phenotypes suggest a defect in terminal differentiation of the seam cells. Although cell differentiation in general was not found to be affected by loss of CKI-1 (van den Heuvel 2005), our results indicate that reduction of function for *cki-1* may interfere with the ability of the seam cells to terminally differentiate properly, perhaps due to failure to exit the cell cycle at the proper time of development. It is currently unclear whether *cdk-4* and *cki-1* are both targets of the Wnt pathway in the same cells at the same time in development; further analysis will be needed to resolve this apparent incongruous result.

#### kin-10 (casein kinase II beta regulatory subunit):

*kin-10* encodes an ortholog of the human casein kinase II beta regulatory subunit encoded by *CSNK2B*. In *C. elegans*, *kin-10* is widely expressed throughout development, although specific expression in seam cells and VPCs was not reported (Hu *et al.* 2006). KIN-10 is required for male mating, general viability, and function of the RNA interference pathway (Kim *et al.* 2005; Hu *et al.* 2006). Interestingly, we observed an increase in terminal seam cell number in *kin-10(RNAi*) animals (Table 3, Figure 3B), and >80% of these animals had misformed seam syncytium and alae (Table 4, Table 5, Figure 3, G and L). These defects were also seen in animals containing the *(ok1751)* mutation, which is a large internal deletion in the *kin-10* gene (Yook *et al.* 2012) (Table 4, Table 5). Finally, as observed previously in a genome-wide RNAi screen (Simmer *et al.* 2003), *kin-10(RNAi)* worms had vulval defects, with 85% of animals having an underinduced phenotype, indicating a possible previously unidentified role for casein kinase II in vulval development (Table 6, Figure 4B).

In *Xenopus* and mice, casein kinase 2 has been shown to be a positive modulator of Wnt/β-catenin signaling and was activated upon Wnt stimulation in murine cell culture (Song *et al.* 2003; Dominguez *et al.* 2004). Some components of the Wnt pathway such as Dvl, APC, TCF, and β-catenin are *in vitro* substrates of CK2 (Dominguez *et al.* 2009). Curiously, our results show that the *kin-10(RNAi)* phenotype in the seam cells is the same as that caused by overactivation of the WBA pathway (an increase in seam cell number) (Gleason and Eisenmann 2010), whereas the *kin-10(RNAi)* phenotype in the vulval cells is the same as that caused by reduction of function of the WBC pathway (underinduction) (Eisenmann *et al.* 1998; Gleason *et al.* 2006). It is possible that *kin-10* may be a target of the WBA pathway in the seam cells and a target of the WBC pathway in the VPCs, and may modulate the function of these pathways in different ways in these two cell types. Finally, the defects in seam cell terminal differentiation seen in *kin-10(RNAi)* animals could be a consequence of the increase in seam cell number or KIN-10 could have separate functions in seam cell division/specification and differentiation. Additional experiments will be needed to characterize the role of KIN-10 in these Wnt-mediated processes.

#### nhr-23 (nuclear hormone receptor 23):

*nhr-23* encodes an orphan nuclear hormone receptor (NHR) that is expressed in the seam cells and the major hypodermal syncytium hyp7 throughout larval development, with expression peaking in the mid larval stage (Kostrouchova *et al.* 1998). Like its fly counterpart DHR3, NHR-23 is involved in molting and may be a regulator of other genes acting in the process (Kostrouchova *et al.* 1998, 2001). We found that RNAi treatment against *nhr-23* starting from the L1 stage resulted in animals with severe molting defects, as reported previously (Kostrouchova *et al.* 1998, 2001). However, we also observed that the seam cell number in *nhr-23(RNAi)* animals was reduced (13.3 SCs/side) (Table 3, Figure 3C), and almost 100% of *nhr-23(RNAi)* animals had defects in seam cell syncytium formation and alae formation (Table 4, Table 5, Figure 3H), indicating a previously unexplored role for NHR-23 in these processes. A "branched" alae defect in *nhr-23(RNAi)* animals was previously noted, although a defect in seam cell number was not seen (Kostrouchova *et al.* 1998, 2001). Since Wnt pathway mutants do not show molting defects (J. Gleason, L. Gorrepati and D. Eisenmann, unpublished observations), it is possible that Wnt signaling may be only one component of the regulation of *nhr-23* expression in the larva, such that reduction of Wnt signaling alone may not be sufficient to cause the molting phenotypes observed in *nhr-23(RNAi)* worms.

#### mlt-11 (molting defective 11):

The process of molting requires spatially coordinated and sequential functioning of transcription factors, signaling molecules, proteases, protease inhibitors, and other factors (Frand *et al.* 2005; Monsalve and Frand 2012). The *mlt-11* gene, which encodes a serine protease inhibitor, was identified in an RNAi screen for molting defects (Frand *et al.* 2005). *mlt-11* is expressed in the seam cells and hypodermis throughout larval development, with a peak of expression in the seam cells right before each larval molt, coinciding with its predicted role in protecting the new cuticle from proteases that are required to degrade the old cuticle (Frand *et al.* 2005). Interestingly, in addition to molting defects, we found that *mlt-11(RNAi)* animals had a decreased number of seam cells as adults (10.5 SCs/side) (Table 3), and a large percentage of *mlt-11(RNAi)* animals showed defects in seam syncytium and alae formation (Table 4, Table 5). This effect on seam cell number was surprising given the proposed extracellular function of MLT-11 in the molting process. We examined 12 other genes identified in molting screens for defects in seam cell number and found that reduction of function for *mlt-7* also caused a decrease in adult cells expressing three different markers of seam cell fate (Table S6). *mlt-7* encodes a heme peroxidase expressed in the hypodermis (including lateral and ventral cells) required for cross-linking of cuticle collagens (Thein *et al.* 2009). Intriguingly, the effect of *mlt-7* and *mlt-11* reduction of function on seam cell number suggests that there may be feedback regulation from the extracellular molting process to the division behavior of the seam cells, as was suggested earlier (Frand *et al.* 2005). Finally, it should be noted that *mlt-11* is predicted to function downstream of NHR-23, as its expression was affected in *nhr-23(RNAi)* animals (Frand *et al.* 2005). Therefore, it is possible that *mlt-11* was identified in this analysis because it is a downstream target of *nhr-23*, which is upregulated upon Wnt pathway overactivation, and that the effect of *nhr-23(RNAi)* on seam cell number is due to the regulation of *mlt-11* by NHR-23.

### Wnt target genes that affect seam cell fate terminal differentiation only

#### bus-8 (bacterially unswollen 8):

BUS-8 is a glycosyl transferase that functions in N-linked glycosylation of proteins (Partridge *et al.* 2008). BUS-8 acts in the embryo during ventral closure when epithelial cells fuse and migrate to enclose the body (Partridge *et al.* 2008). Postembryonically, BUS-8 is expressed exclusively in the seam cells, where it functions in molting and in maintaining cuticle integrity (Gravato-Nobre *et al.* 2005; Partridge *et al.* 2008). Consistent with previous results, we observed that treatment of *rrf-3* worms with *bus-8* RNAi from the L1 stage resulted in animals with molting defects, some of which developed normally until adulthood and laid eggs that did not hatch. We found that terminal seam cell number in *bus-8(RNAi)* animals was unaffected (data not shown); however, 34% of *bus-8(RNAi)* animals showed gaps in the seam syncytium or had unfused seam cells (Table 4), and 92% of *bus-8(RNAi)* of animals had either partial or no alae (Table 5). These results indicate a previously unidentified role for this protein glycosylation factor in seam cell terminal differentiation. Because *bar-1* loss-of-function mutants do not have seam cell terminal differentiation defects of this type (J. Gleason, L. Gorrepati, and D. Eisenmann, unpublished results), this again suggests that regulation by Wnt signaling may represent only one component of *bus-8* expression, such that loss of Wnt signaling does not phenocopy the *bus-8* mutant seam terminal differentiation phenotype.

#### K10D6.2:

*K10D6.2*, which encodes a claudin-like transmembrane protein, was identified as a gene expressed in *C. elegans* sensory neurons by the mRNA tagging method, and it was also found to be strongly expressed in the seam cells during larval life (Kunitomo *et al.* 2005; Simske and Hardin 2011). In two genome-wide screens, RNAi for *K10D6.2* resulted in embryonic lethality (Maeda *et al.* 2001; Rual *et al.* 2004). As with *bus-8*, *K10D6.2(RNAi)* animals showed no significant change in adult seam cell number (data not shown), but 100% of adults had defects in formation of the adult seam syncytium, with unfused seam cells visible (Table 4, Figure 3I), and 65% of *K10D6.2(RNAi)* adult animals had partial or no alae (Table 5). Even in younger *K10D6.2(RNAi)* larvae, seam cells failed to contact each other to form a linear array, exhibiting gaps along the length of the animal (data not shown). These RNAi results indicate the *K10D6.2* gene product likely functions in seam cell terminal differentiation, perhaps in the homotypic fusion of the seam cells.

### Wnt target genes that act in vulval development

#### pak-1 (p21-activated kinase 1):

*pak-1* encodes a p21 activated kinase homolog required for proper axonal guidance (Lucanic *et al.* 2006), which is expressed in several tissues, including the seam cell T and its descendants, and the VPCs (Iino and Yamamoto 1998; Goh *et al.* 2012). In addition to its role in the specification of VPC fates, Wnt signaling also regulates the polarity of the descendants of secondary-fated VPC, P7.p (Inoue *et al.* 2004; Deshpande *et al.* 2005; Green *et al.* 2007, 2008). Consistent with a role in that process, we found that 8% of *pak-1(RNAi)* animals had a Bivulva phenotype indicative of P7.p polarity defects (Table 6). This phenotype was slightly stronger in *pak-1(ok448)* animals, which carry a large deletion in the *pak-1* gene (Harris *et al.* 2010; Yook *et al.* 2012) (Table 6). Curiously, others have reported that only ∼1% of *pak-1(ok448)* mutant worms exhibited defects in P7.p polarity, but that *pak-1(ok448)* could suppress the defects in P7.p polarity and reporter gene expression exhibited by a mutation in *lin-17*, which encodes a member of the Frizzled family of Wnt receptors (Goh *et al.* 2012). Therefore, it was proposed that PAK-1 negatively regulates the Wnt signaling pathway acting in P7.p polarity during vulval development (Goh *et al.* 2012). Our results show that loss of function of *pak-1* by RNAi or mutation results in a weak Bivulva phenotype similar to that caused by reduction of Wnt signaling, suggesting that PAK-1 plays a positive role downstream of Wnt signaling in P7.p. It is not clear why there is a difference in results, although differences in strain background could be possible. PAK-1 was recently shown to regulate the phosphorylation of β-catenin in colon cancer cells (Zhu *et al.* 2012). It is possible that *pak-1* may be a downstream target of Wnt signaling in vulval development that is required for phosphorylation of Wnt signaling components or other proteins necessary for successful orientation of P7.p polarity.

#### lin-1 (Lineage abnormal 1):

*lin-1* encodes an Ets-domain transcription factor that acts downstream of RTK/Ras signaling in the VPC P6.p during vulval development (Beitel *et al.* 1995). Loss of function of *lin-1* causes induction of VPCs, resulting in a multivulva phenotype, suggesting LIN-1 prevents these VPCs from adopting induced vulval cell fates in the absence of RTK/Ras signaling (Beitel *et al.* 1995; Sternberg 2005). Consistent with this, we found that 91% of *lin-1(RNAi)* animals had ectopic vulval invaginations characteristic of a multivulva phenotype (Table 6). Because the overinduced vulval phenotype caused by loss of *lin-1* activity is the opposite of the underinduced phenotype resulting from reduction of Wnt pathway activity (Eisenmann *et al.* 1998; Eisenmann and Kim 2000; Gleason *et al.* 2006), it was intriguing to identify *lin-1* as a possible Wnt pathway target. qPCR showed that endogenous *lin-1* transcript levels increased slightly upon Wnt pathway activation and decreased slightly upon Wnt pathway inhibition (Table S5). Therefore, we hypothesize that Wnt signaling may be one of several components that maintain *lin-1* expression in the VPCs before induction, such that while overexpression of ΔNTBAR-1 upregulates *lin-1* expression, reduction of Wnt signaling, such as in a *bar-1(ga80)* mutant, does not reduce *lin-1* expression enough to cause an overinduction phenotype.

## Conclusion

Forward genetic analysis in *C. elegans* has proven very useful in identifying signaling pathways regulating many developmental processes (Greenwald 2005). However, the same forward genetic techniques have often proven much less effective at identifying the targets of these pathways that actually effect the transcription changes underlying the biological responses elicited by pathway activation. A possible explanation for this result is that inactivation of a single target gene may be unlikely to cause a strong phenotype like that caused by loss of core signaling pathway components. Therefore, genomic approaches may be useful for identifying the downstream targets of signaling pathways acting during development.

We previously showed that Wnt signaling plays a role in cell fate specification of hypodermal seam and vulval precursor cells during *C. elegans* larval development (Eisenmann *et al.* 1998; Gleason *et al.* 2002, 2006; Gleason and Eisenmann 2010). However, few direct targets of the Wnt pathway in these cells have been identified (Jackson and Eisenmann 2012). To attempt to identify Wnt pathway target genes in *C. elegans*, we used a gain-of-function approach, in which we conditionally expressed a gain-of-function BAR-1 beta catenin protein at the time of the L2/L3 molt, followed by microarray analysis to identify differentially regulated genes. In our initial approach, we analyzed changes in gene expression from the whole animal and identified more than 100 genes that showed increased expression upon delNTBAR-1 expression (Jackson *et al.* 2014).

Here, we combined this previous approach with the "mRNA tagging" method (Roy *et al.* 2002) to enrich for transcripts from the seam and vulval precursor cells. We believe this combination of approaches has been successful at finding cell type–specific targets of the Wnt signaling pathway because we identified many genes known to be expressed in or function in the seam or vulval precursor cells, and we showed that nine of the genes identified by this method have loss-of-function phenotypes consistent with a function in these cell types. Several of these genes were known to function in hypodermal cell division or to have phenotypes affecting the seam or vulval precursor cells; however, a number of genes (*bus-8*, *cki-1*, *K10D6.2*, *kin-10*, *mlt-11*, *nhr-23)* were shown to have novel phenotypes in one or both of these cell types upon reduction of gene function. Further, in work reported elsewhere, we showed that the GATA factor encoding genes *egl-18* and *elt-6* identified by this method are targets of the Wnt pathway in the seam cells and are required for the maintenance of the seam cell "progenitor" fate in one daughter of the larval seam cell asymmetric divisions (Gorrepati *et al.* 2013). Given the success of this method in identifying Wnt target genes acting in the larval hypodermal cells, an approach like this could be useful for identifying target genes regulated by other signaling pathways that function in *C. elegans* development, if conditional pathway activation using suitable reagents is possible.

We chose the approach of conditionally expressing a gain-of-function beta catenin variant over the more simple approach of analyzing gene expression in a Wnt pathway loss-of-function strain as a way to bypass any indirect effects on gene expression that might result from cell fate transformations due to loss of Wnt pathway activity (Jackson *et al.* 2014). Intriguingly, a recent report showed that a large number of genes show alterations in expression at the L4 stage in a strain containing a *bar-1* loss-of-function mutation **(**van der Bent *et al.* 2014). The fact that a small set of genes showed reduced expression upon loss of *bar-1* (van der Bent *et al.* 2014) and increased expression upon expression of activated BAR-1 (Jackson *et al.* 2014; this work) identifies these gene as strong candidates for *bona fide* BAR-1-regulated or Wnt-regulated target genes. Therefore, the use of complementary loss-of-function and gain-of-function approaches, when possible, may strongly highlight a subset of genes as targets of a signaling pathway for further in-depth biological investigation.

## 

## Supplementary Material

Supporting Information
