# Supplementary material for: Identification of Wnt Pathway Target Genes Regulating the Division and Differentiation of Larval Seam Cells and Vulval Precursor Cells in Caenorhabditis elegans
Source: G3 (Bethesda). 2015 Jun 5;5(8):1551–66. doi: 10.1534/g3.115.017715 (PMC4528312; doi:10.1534/g3.115.017715)
Supplement: Supporting Information [file supp_5_8_1551__index.html]

Identification of Wnt Pathway Target Genes Regulating the Division and Differentiation of Larval Seam Cells and Vulval Precursor Cells in Caenorhabditis elegans — Supporting Information 

# Identification of Wnt Pathway Target Genes Regulating the Division and Differentiation of Larval Seam Cells and Vulval Precursor Cells in *Caenorhabditis elegans*

## Supporting Information for Gorrepati *et al.*, 2015

**Files in this Data Supplement:**

- Supporting Information - Figures S1-S2 and Tables S1-S7 (PDF, 249 KB)
- Figure S1 - mRNA tagging method requires FLAG-PAB-1 expression in seam cells and VPCs. (PDF, 83 KB)
- Figure S2 - Lineage of larval seam cells. (PDF, 151 KB)
- Table S1 - 239 putative Wnt targets from seam cells and VPCs. (PDF, 84 KB)
- Table S2 - 24 putative Wnt target genes in common between Jackson et al., 2014 and this work. (PDF, 66 KB)
- Table S3 - Genes in common between van der Bent et al., 2014 and this work. (PDF, 69 KB)
- Table S4 - DAVID analysis identifies four functionally related groups among cell type specific Wnt targets. (PDF, 95 KB)
- Table S5 - 27 seam cell/VPC Wnt regulated genes validated by qPCR. (PDF, 79 KB)
- Table S6 - Reduction of function for molting genes *mlt-7* and *mlt-11* causes a reduction in terminal seam cell number. (PDF, 68 KB)
- Table S7 - Oligonucleotide sequences. (.xls, 16 KB)
