# Supplementary material for: Identification of Wnt Pathway Target Genes Regulating the Division and Differentiation of Larval Seam Cells and Vulval Precursor Cells in Caenorhabditis elegans
Source: G3 (Bethesda). 2015 Jun 5;5(8):1551–66. doi: 10.1534/g3.115.017715 (PMC4528312; doi:10.1534/g3.115.017715)
Supplement: Supporting Information [file supp_g3.115.017715_017715SI.pdf]

G3: Genes | Genomes | Genetics

Identification of Wnt pathway target genes regulating the division and differentiation of larval seam cells and vulval precursor cells in *C. elegans*

Lakshmi Gorrepati[§1], Michael W. Krause[*], Weiping Chen[*], Thomas M. Brodigan[*], Margarita Correa-Mendez[§], and David M. Eisenmann[§2]

Author Affiliations

* Intramural Research Program, NIDDK, Bethesda, MD, 20814

§ Department of Biological Sciences, University of Maryland Baltimore County, Baltimore, 21250

Present address

1 Department of Embryology, Carnegie Institute for Science, Baltimore, MD, 21218

Microarray data accession number: GSE52747

2 Corresponding author

David M. Eisenmann

Department of Biological Sciences,

University of Maryland Baltimore County

1000 Hilltop Circle, Baltimore, MD, 21250

Ph: 410-455-2256

Fax:

Email: eisenman@umbc.edu

DOI: 10.1534/g3.115.017715

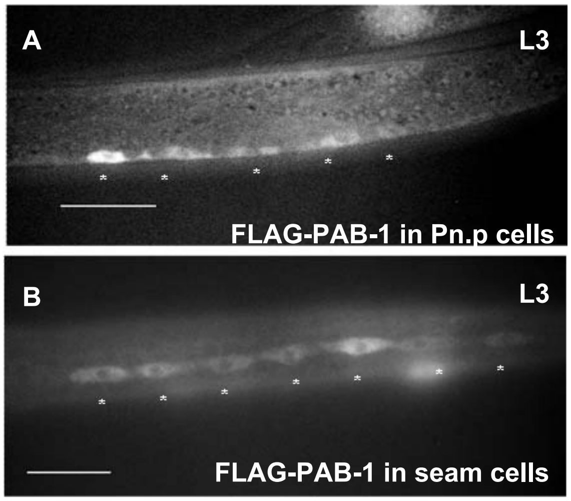

Figure S1   mRNA tagging method requires FLAG-PAB-1 expression in seam cells and VPCs

FLAG-PAB-1 expressed in the L2/L3 worms can be seen in the VPCs (A) and seam cells (B) of the experimental strain *deIs10; scm::gfp* by antibody staining against the FLAG epitope. The asterisks mark the stained cells. Scale bars represent 50μm.

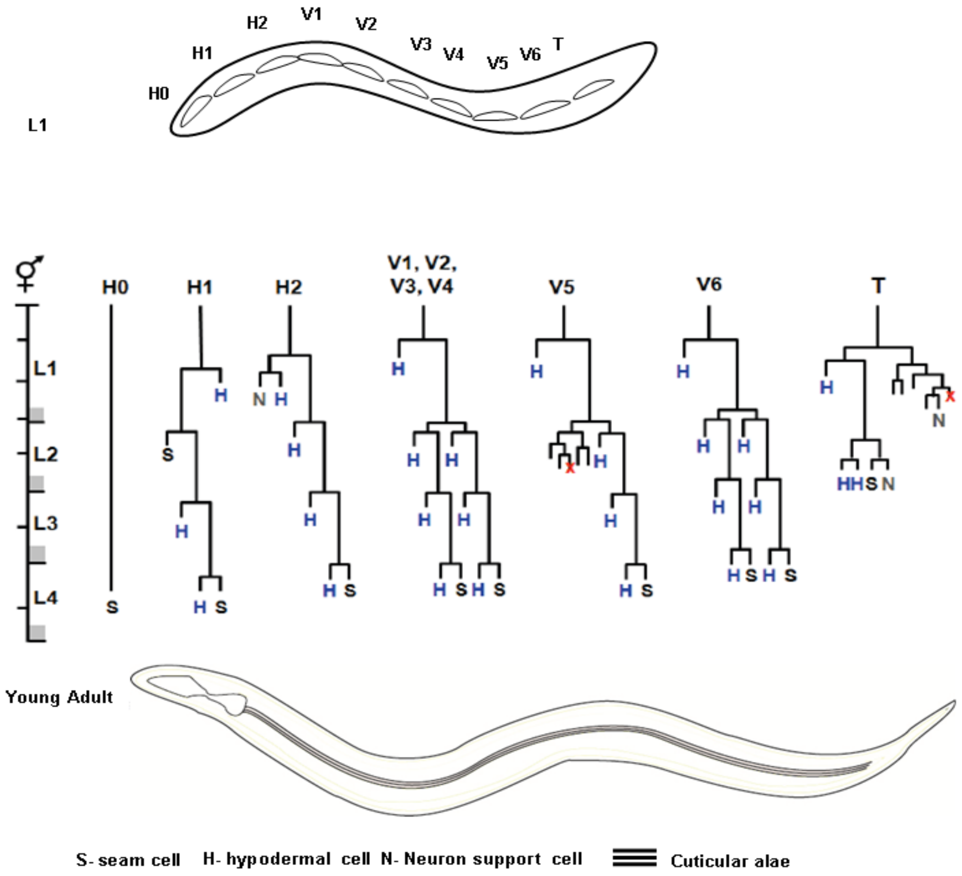

Figure S2 Lineage of larval seam cells

An L1 worm has 10 seam cells. At L2, six seam cells undergo a proliferative division to increase the number of seam cells to 16. In each larval stage, most seam cells divide asymmetrically to maintain their population as well as contribute to the hypodermal syncytium and to other neuronal support structures. After their final division, seam cells differentiate and undergo homotypic fusion to form a single-celled syncytium which secretes a specialized cuticular structure called alae.

Table S1 239 putative Wnt targets from seam cells and VPCs. Shown are 239 putative Wnt targets identified by microarray analysis: the average fold change and p value in Wnt pathway over-activation conditions is shown for each gene. '*' indicates 24 genes previously known to be expressed in the seam cells (Wormbase release 220). '§' indicates 12 genes previously known to be expressed in the vulval cell lineage (Wormbase release 220) '#' indicates five genes shown to be expressed in seam cells, hypodermal cells and P cells by Jackson et al., 2014.

Gene WB ID	Gene Public Name	p-value Wnt over-activation	fold change Wnt Over-activation
**WBGene00000615**	***col-38*[#]**	**0.000**	**29.7**
**WBGene00000626**	***col-49*[#]**	**0.000**	**23.5**
**WBGene00013754**	***fbxa-116***	**0.002**	**16.1**
**WBGene00006509**	***tag-164***	**0.000**	**14.6**
**WBGene00007381**	***C06C6.7***	**0.000**	**14.3**
**WBGene00018295**	***oac-29***	**0.002**	**14.2**
**WBGene00012046**	***T26E4.4*[#]**	**0.003**	**13.3**
**WBGene00014079**	***ZK792.7***	**0.000**	**12.8**
**WBGene00022140**	***Y71G12A.4***	**0.008**	**12.1**
**WBGene00009898**	***dod-23***	**0.000**	**11.8**
**WBGene00013986**	***ZK512.7***	**0.001**	**11.2**
**WBGene00001068**	***dpy-6***	**0.004**	**10.7**
**WBGene00015765**	***C14C11.1***	**0.000**	**10.3**
**WBGene00022589**	***ZC317.6***	**0.000**	**10.3**
**WBGene00008211**	***C49F5.7***	**0.002**	**10.3**
**WBGene00016058**	***nspd-3***	**0.001**	**10.0**
**WBGene00012783**	***Y43C5A.3***	**0.001**	**10.0**
**WBGene00003763**	***nlp-25***	**0.017**	**10.0**
**WBGene00044630**	***bus-17***	**0.007**	**9.6**
**WBGene00017485**	***F15E6.4***	**0.001**	**9.4**
**WBGene00017488**	***dct-7***	**0.031**	**8.7**
**WBGene00016683**	***C45G9.12***	**0.004**	**8.5**
**WBGene00011226**	***R11.1***	**0.001**	**7.8**
**WBGene00011624**	***T08G5.3***	**0.004**	**7.8**
**WBGene00021379**	***Y37E11B.7***	**0.034**	**7.7**
**WBGene00012591**	***nspe-1***	**0.022**	**7.5**
**WBGene00015182**	***B0416.7***	**0.002**	**7.4**
**WBGene00001691**	***grd-2***	**0.004**	**6.9**
**WBGene00003862**	***old-1***	**0.004**	**6.7**
**WBGene00003863**	***old-2***	**0.004**	**6.7**
**WBGene00018380**	***F43C11.3***	**0.036**	**6.6**
**WBGene00021731**	***Y49G5A.1***	**0.000**	**6.3**
**WBGene00000647**	***col-71*[#]**	**0.001**	**6.2**
**WBGene00020033**	***R12E2.7*[§*]**	**0.012**	**6.2**
**WBGene00017759**	***F23H11.3***	**0.002**	**6.0**
**WBGene00008472**	***E03H4.4***	**0.000**	**6.0**
**WBGene00013514**	***Y73F4A.1***	**0.019**	**6.0**

**WBGene00018744**	***F53B3.6***	**0.008**	**5.9**
**WBGene00022024**	***Y64H9A.2***	**0.000**	**5.9**
**WBGene00009130**	***F25H5.8***	**0.012**	**5.8**
**WBGene00000656**	***col-80***	**0.007**	**5.8**
**WBGene00001079**	***dpy-20***	**0.002**	**5.6**
**WBGene00007297**	***C04F12.1***	**0.004**	**5.5**
**WBGene00050896**	***ttr-56***	**0.000**	**5.4**
**WBGene00010856**	***M04C9.1***	**0.001**	**5.4**
**WBGene00010743**	***K10D6.3***	**0.012**	**5.2**
**WBGene00001882**	***his-8***	**0.001**	**5.2**
**WBGene00001894**	***his-20***	**0.001**	**5.2**
**WBGene00001896**	***his-22***	**0.001**	**5.2**
**WBGene00001926**	***his-52***	**0.001**	**5.2**
**WBGene00011077**	***R07B1.5***	**0.007**	**5.2**
**WBGene00004394**	***rol-1***	**0.001**	**5.2**
**WBGene00021427**	***Y38F2AR.9***	**0.006**	**5.2**
**WBGene00015758**	***nhr-155***	**0.001**	**5.1**
**WBGene00000930**	***dao-4***[#]	**0.011**	**5.0**
**WBGene00018219**	***F40A3.4***	**0.003**	**5.0**
**WBGene00015865**	***C16E9.1***	**0.004**	**4.9**
**WBGene00016926**	***nhr-171***	**0.005**	**4.8**
**WBGene00011948**	***T23F1.5***	**0.000**	**4.8**
**WBGene00003664**	***nhr-74****	**0.004**	**4.8**
**WBGene00011814**	***T16H12.4***	**0.011**	**4.8**
**WBGene00045387**	***K09H9.8***	**0.004**	**4.7**
**WBGene00021994**	***Y59E9AL.4***	**0.005**	**4.7**
**WBGene00011533**	***T06E4.5***	**0.010**	**4.6**
**WBGene00020380**	***T09B4.6***	**0.006**	**4.5**
**WBGene00020769**	***T24D8.6***	**0.020**	**4.5**
**WBGene00021605**	***Y46H3C.5***	**0.013**	**4.5**
**WBGene00015172**	***B0410.3***	**0.001**	**4.5**
**WBGene00014003**	***ZK593.3***	**0.050**	**4.5**
**WBGene00011023**	***R05A10.6***	**0.014**	**4.5**
**WBGene00015769**	***C14C11.7***	**0.001**	**4.5**
**WBGene00013438**	***ztf-29***	**0.030**	**4.4**
**WBGene00003703**	***nhr-113****	**0.019**	**4.3**
**WBGene00015340**	***C02E7.7***	**0.000**	**4.3**
**WBGene00006534**	***tba-8****	**0.001**	**4.3**
**WBGene00012594**	***nspe-5***	**0.000**	**4.3**
**WBGene00004202**	***pry-1***[§*]	**0.018**	**4.2**
**WBGene00017436**	***F13H8.3***	**0.029**	**4.1**
**WBGene00020613**	***T20D4.7***	**0.036**	**4.1**
**WBGene00000493**	***che-14***[§*]	**0.037**	**4.1**
**WBGene00022816**	***fbn-1***	**0.027**	**4.1**
**WBGene00043147**	***nspd-6***	**0.004**	**4.1**
**WBGene00015339**	***C02E7.6***	**0.000**	**4.1**
**WBGene00009026**	***F21G4.5***	**0.008**	**4.0**

**WBGene00000406**	***cdk-4***[§*]	**0.007**	**3.9**
**WBGene00005672**	***sru-9***	**0.000**	**3.9**
**WBGene00011594**	***T07G12.3***	**0.033**	**3.9**
**WBGene00001885**	***his-11***	**0.005**	**3.9**
**WBGene00001889**	***his-15***	**0.005**	**3.9**
**WBGene00001918**	***his-44****	**0.005**	**3.9**
**WBGene00019306**	***K02E7.1***	**0.043**	**3.9**
**WBGene00018416**	***retr-1***	**0.007**	**3.9**
**WBGene00009787**	***F46F2.3***	**0.018**	**3.8**
**WBGene00000516**	***cki-1***[§*]	**0.007**	**3.8**
**WBGene00015196**	***B0454.5***	**0.011**	**3.8**
**WBGene00000072**	***add-1****	**0.009**	**3.7**
**WBGene00011033**	***R05D11.5***	**0.000**	**3.7**
**WBGene00017297**	***F09E10.7***	**0.002**	**3.7**
**WBGene00000653**	***col-77***	**0.001**	**3.7**
**WBGene00013661**	***Y105C5B.23***	**0.031**	**3.7**
**WBGene00001878**	***his-4***	**0.003**	**3.7**
**WBGene00002393**	***lpr-1***	**0.020**	**3.6**
**WBGene00017799**	***F25G6.8***	**0.015**	**3.6**
**WBGene00012324**	***rhy-1***	**0.029**	**3.6**
**WBGene00009590**	***ttr-4***	**0.012**	**3.6**
**WBGene00010538**	***ttr-3***	**0.012**	**3.6**
**WBGene00015051**	***B0218.7***	**0.000**	**3.6**
**WBGene00006367**	***sym-2***	**0.001**	**3.6**
**WBGene00003446**	***msp-53***	**0.002**	**3.5**
**WBGene00006539**	***tbb-6***	**0.000**	**3.5**
**WBGene00020873**	***T28A11.6***	**0.027**	**3.5**
**WBGene00011665**	***T09F5.1***	**0.010**	**3.5**
**WBGene00009160**	***F26E4.5***	**0.018**	**3.5**
**WBGene00000711**	***col-138***	**0.000**	**3.4**
**WBGene00011398**	***qdpr-1***	**0.021**	**3.4**
**WBGene00017783**	***F25E5.2***	**0.008**	**3.4**
**WBGene00021997**	***Y59E9AR.1***	**0.001**	**3.4**
**WBGene00022002**	***Y59E9AR.7***	**0.001**	**3.4**
**WBGene00021927**	***Y55F3AM.10***	**0.001**	**3.4**
**WBGene00016919**	***C54E4.4***	**0.025**	**3.4**
**WBGene00015442**	***C04F1.1***	**0.050**	**3.4**
**WBGene00016641**	***C44C1.1***	**0.014**	**3.3**
**WBGene00007770**	***nhr-259***	**0.038**	**3.3**
**WBGene00018689**	***F52D2.7***	**0.001**	**3.3**
**WBGene00015987**	***C18G1.9***	**0.001**	**3.3**
**WBGene00012323**	***oac-54***	**0.032**	**3.3**
**WBGene00018702**	***F52E4.5***	**0.002**	**3.2**
**WBGene00001984**	***hog-1***	**0.018**	**3.1**
**WBGene00019234**	***ugt-8***	**0.011**	**3.1**

WBGene00000596	*col-7**	0.010	3.1
WBGene00000638	*col-62*	0.010	3.1
WBGene00003102	*mab-5*[§*]	0.013	3.1
WBGene00008290	*C54C8.2**	0.033	3.1
WBGene00020216	*trap-2*	0.031	3.1
WBGene00020445	*T12B3.2*	0.008	3.1
WBGene00015950	*C18A11.4*	0.000	3.1
WBGene00010225	*ttr-31*	0.025	3.0
WBGene00010800	*srsx-37*	0.009	3.0
WBGene00012055	*srsx-36*	0.009	3.0
WBGene00016192	*C28H8.1*	0.002	3.0
WBGene00000734	*col-161*	0.008	3.0
WBGene00000735	*col-162*	0.008	3.0
WBGene00001480	*fmo-5*	0.015	3.0
WBGene00014162	*ZK938.6*	0.006	3.0
WBGene00016294	*C31H2.4*	0.008	2.9
WBGene00044623	*bus-8**	0.013	2.9
WBGene00015453	*C04G6.2*	0.007	2.9
WBGene00077489	*C04G6.13*	0.007	2.9
WBGene00003444	*msp-51*	0.026	2.9
WBGene00003448	*msp-55*	0.026	2.9
WBGene00003450	*msp-57*	0.026	2.9
WBGene00011434	*T04D3.5*	0.016	2.8
WBGene00006660	*twk-5*	0.015	2.8
WBGene00000969	*dhs-5*	0.013	2.8
WBGene00001253	*elt-6*[§]	0.011	2.8
WBGene00021486	*lbp-9*	0.001	2.8
WBGene00010742	*K10D6.2*	0.004	2.7
WBGene00016449	*C35D10.13*	0.002	2.7
WBGene00003622	*nhr-23*	0.002	2.7
WBGene00000720	*col-147*	0.050	2.7
WBGene00008435	*glna-2*	0.022	2.6
WBGene00016011	*C23G10.2*	0.007	2.6
WBGene00012165	*cutl-6*	0.049	2.6
WBGene00017348	*F10E7.6*	0.036	2.6
WBGene00009657	*F43G6.4*	0.023	2.6
WBGene00001692	*grd-3**	0.008	2.5
WBGene00012420	*Y7A9D.1*	0.007	2.5
WBGene00009489	*F36G9.13*	0.001	2.5
WBGene00009675	*F44A6.3*	0.034	2.5
WBGene00002070	*ile-1*	0.004	2.5
WBGene00000990	*dhs-27*	0.016	2.5
WBGene00016346	*fbxa-120*	0.006	2.5
WBGene00018252	*F40H3.6*	0.003	2.5
WBGene00012364	*W09D12.1*	0.009	2.5

**WBGene00001703**	***grd-14****	**0.002**	**2.4**
**WBGene00010289**	***F58H1.6***	**0.015**	**2.4**
**WBGene00021845**	***rpb-7***	**0.000**	**2.4**
**WBGene00007592**	***C14H10.2***	**0.015**	**2.4**
**WBGene00012443**	***Y15E3A.4***	**0.010**	**2.3**
**WBGene00009807**	***pepm-1***	**0.004**	**2.3**
**WBGene00015593**	***C08E3.1***	**0.036**	**2.3**
**WBGene00015605**	***C08E3.13***	**0.036**	**2.3**
**WBGene00000601**	***col-12***	**0.028**	**2.3**
**WBGene00003611**	***nhr-12***	**0.015**	**2.3**
**WBGene00005025**	***sqv-7*** [§*]	**0.007**	**2.2**
**WBGene00017082**	***DC2.5***	**0.004**	**2.2**
**WBGene00010051**	***F54D5.5***	**0.018**	**2.2**
**WBGene00011890**	***T21C9.1***	**0.005**	**2.2**
**WBGene00007925**	***C34C12.6***	**0.022**	**2.1**
**WBGene00006476**	***rhgf-2***	**0.009**	**2.1**
**WBGene00000532**	***clh-5***	**0.031**	**2.1**
**WBGene00000602**	***col-13***	**0.036**	**2.1**
**WBGene00019666**	***sago-1*** [§*]	**0.024**	**2.1**
**WBGene00016929**	***C54F6.12***	**0.024**	**2.1**
**WBGene00019331**	***dos-3***	**0.008**	**2.1**
**WBGene00004272**	***rab-8***	**0.045**	**2.1**
**WBGene00044349**	***Y71G12B.32***	**0.000**	**2.1**
**WBGene00000729**	***col-156***	**0.027**	**2.1**
**WBGene00013981**	***ZK507.6***	**0.036**	**2.1**
**WBGene00010470**	***cdr-4***	**0.027**	**2.1**
**WBGene00013292**	***Y57G11A.4***	**0.017**	**2.0**
**WBGene00011561**	***ttr-15***	**0.009**	**2.0**
**WBGene00001073**	***dpy-11***	**0.026**	**2.0**
**WBGene00021789**	***nol-6***	**0.017**	**2.0**
**WBGene00010523**	***K03B8.4***	**0.040**	**2.0**
**WBGene00001971**	***hmg-1.1*** [§*]	**0.022**	**2.0**
**WBGene00019826**	***R02E4.1***	**0.004**	**2.0**
**WBGene00002990**	***lin-1***	**0.038**	**1.9**
**WBGene00018488**	***acs-1***	**0.048**	**1.9**
**WBGene00004164**	***pqn-83***	**0.015**	**1.9**
**WBGene00004702**	***rsp-5***	**0.004**	**1.9**
**WBGene00002261**	***ldb-1***	**0.047**	**1.9**
**WBGene00006543**	***tbx-2***	**0.039**	**1.9**
**WBGene00008571**	***F08B12.1***	**0.040**	**1.9**
**WBGene00020109**	***R151.6***	**0.013**	**1.9**
**WBGene00012186**	***mlt-11****	**0.043**	**1.8**
**WBGene00020263**	***T05E8.3***	**0.010**	**1.8**
**WBGene00011731**	***acbp-5***	**0.037**	**1.8**
**WBGene00018341**	***F42A10.5***	**0.049**	**1.8**

**WBGene00000100**	***ajm-1*§***	**0.026**	**1.8**
**WBGene00000730**	***col-157***	**0.023**	**1.8**
**WBGene00000728**	***col-155***	**0.030**	**1.8**
**WBGene00004701**	***rsp-4****	**0.006**	**1.7**
**WBGene00000727**	***col-154***	**0.029**	**1.7**
**WBGene00022164**	***Y71H2AL.1***	**0.042**	**1.7**
**WBGene00014186**	***nhr-244***	**0.019**	**1.7**
**WBGene00002051**	***ifa-3***	**0.004**	**1.7**
**WBGene00019850**	***srt-18***	**0.019**	**1.7**
**WBGene00009381**	***F34H10.4***	**0.023**	**1.7**
**WBGene00019237**	***H24G06.1***	**0.021**	**1.7**
**WBGene00001928**	***his-54***	**0.042**	**1.7**
**WBGene00003911**	***pak-1***	**0.025**	**1.6**
**WBGene00010317**	***idh-1*§***	**0.044**	**1.6**
**WBGene00003891**	***osm-11*§***	**0.047**	**1.6**
**WBGene00015757**	***C14C6.3***	**0.009**	**1.6**
**WBGene00014098**	***ZK836.2***	**0.022**	**1.6**
**WBGene00011604**	***T08A11.1***	**0.029**	**1.5**
**WBGene00015181**	***B0416.5***	**0.003**	**1.5**
**WBGene00002196**	***kin-10***	**0.124**	**1.5**

Table S2 24 putative Wnt target genes in common between Jackson et al., 2014 and this work. Of the 239 putative Wnt pathway regulated genes from seam cells and VPCs identified here by mRNA tagging, 24 genes were also identified by Jackson et al., 2014 in a non-tissue specific analysis of Wnt upregulated genes.

Gene WB ID	Gene
WBGene00000615	*col-38*
WBGene00000626	*col-49*
WBGene00000647	*col-71*
WBGene00000930	*dao-4*
WBGene00001984	*hog-1*
WBGene00004202	*pry-1*
WBGene00006509	*tag-164*
WBGene00006539	*tbb-6*
WBGene00008472	*E03H4.4*
WBGene00009898	*dod-23*
WBGene00010470	*cdr-4*
WBGene00011077	*R07B1.5*
WBGene00011594	*T07G12.3*
WBGene00011624	*T08G5.3*
WBGene00012046	*T26E4.4*
WBGene00012783	*Y43C5A.3*
WBGene00013754	*fbxa-116*
WBGene00015442	*C04F1.1*
WBGene00016058	*nspd-3*
WBGene00017485	*F15E6.4*
WBGene00018295	*oac-29*
WBGene00018416	*retr-1*
WBGene00019234	*ugt-8*
WBGene00043147	*nspd-6*

Table S3 Genes in common between van der Bent et al., 2014 and this work. Of the 239 putative Wnt pathway regulated genes from seam cells and VPCs identified here by mRNA tagging, 35 genes were also identified by van der Bent et al., 2014 as genes that were differentially expressed between a wild type strain and a strain carrying a loss-of-function mutation in *bar-1*. The third column indicates whether expression of the gene was decreased or increased in the *bar-1(ga80)* strain relative to wild type. Genes with an asterisk (*) were identified as downregulated upon loss of *bar-1* in van der Bent et al. 2014 and upregulated upon expression of an activated BAR-1 protein in this work and in Jackson et al . 2014.

Gene WB ID	Gene	*bar-1(ga80)* expression
WBGene00000493	*che-14*	decreased
WBGene00000615	*col-38**	decreased
WBGene00000626	*col-49**	decreased
WBGene00000647	*col-71**	decreased
WBGene00000711	*col-138**	decreased
WBGene00000735	*col-162*	decreased
WBGene00000930	*dao-4**	decreased
WBGene00001073	*dpy-11**	decreased
WBGene00001691	*grd-2**	decreased
WBGene00001885	*his-11*	decreased
WBGene00001984	*hog-1**	decreased
WBGene00002393	*lpr-1*	decreased
WBGene00004394	*rol-1*	decreased
WBGene00011077	*R07B1.5**	decreased
WBGene00011594	*T07G12.3**	decreased
WBGene00011665	*T09F5.1*	decreased
WBGene00012186	*mlt-11*	decreased
WBGene00013514	*Y73F4A.1*	decreased
WBGene00015172	*B0410.3*	decreased
WBGene00015442	*C04F1.1**	decreased
WBGene00015453	*C04G6.2*	decreased
WBGene00015950	*C18A11.4**	decreased
WBGene00017783	*F25E5.2**	decreased
WBGene00022024	*Y64H9A.2*	decreased
WBGene00077489	*C04G6.13*	decreased
WBGene00000720	*col-147*	increased
WBGene00006539	*tbb-6*	Increased
WBGene00007297	*C04F12.1*	Increased
WBGene00010051	*F54D5.5*	Increased
WBGene00011434	*T04D3.5*	Increased
WBGene00011561	*ttr-15*	Increased
WBGene00012783	*Y43C5A.3*	Increased
WBGene00015865	*C16E9.1*	Increased
WBGene00016919	*C54E4.4*	Increased
WBGene00021379	*Y37E11B.7*	increased

Table S4 DAVID analysis identifies four functionally related groups among cell type specific Wnt targets. DAVID analysis of the seam cell/VPC Wnt regulated genes identified four functionally related gene groups. The microarray fold change in the Wnt over-activation condition for each gene is indicated. The enrichment score for each gene group is indicated in parenthesis (column 2). The enrichment score is the geometric mean of the modified Fisher's exact p-value (EASE score) of each gene in a given gene group. A higher enrichment score indicates a higher rank in functional importance (Huang et al. 2009a, 2009b). Of these 57 genes grouped by DAVID, seven genes are expressed in seam (indicated by '#', column 2), two genes in the vulva (indicated by 'Δ') and five in the seam and vulva (indicated by '§') (Wormbase release 220). '*' indicates genes in this list that were characterized further in this work.

Gene	Gene description	fold change Wnt over-activation
Gene group 1	***Structural constituents of cuticle (enrichment score 7.3)***	
che-14*	**abnormal CHEmotaxis§**	**4.1**
col-7	**COLlagen#**	**3.1**
col-12	**COLlagen**	**2.3**
col-13	**COLlagen**	**2.1**
col-38	**COLlagen**	**29.7**
col-49*	**COLlagen**	**23.5**
col-62	**COLlagen**	**3.1**
col-71	**COLlagen**	**6.2**
col-77	**COLlagen**	**3.7**
col-80	**COLlagen**	**5.8**
col-138	**COLlagen**	**3.4**
col-147	**COLlagen**	**2.7**
col-154	**COLlagen**	**1.7**
col-155	**COLlagen**	**1.8**
col-156	**COLlagen**	**2.1**
col-157	**COLlagen**	**1.8**
col-161	**COLlagen**	**3.0**
col-162	**COLlagen**	**3.0**
pqn-83	**Prion-like-(Q/N-rich)-domain protein**	**1.9**
rol-1	**ROLler/collagen**	**5.2**
F54D5.5	**F54D5.5**	**2.2**
K03B8.4	**K03B8.4**	**2.0**
T04D3.5	**T04D3.5**	**2.8**
T06E4.5	**T06E4.5**	**4.6**
C44C1.1	**C44C1.1**	**3.3**
Gene group 2	***Transcription factors (enrichment score 0.64)***	
dpy-20	**DumPY**	**5.6**
elt-6*	**Erythroid-Like Transcription factor family§**	**2.8**
nhr-12	**Nuclear Hormone Receptor family**	**2.3**
nhr-23*	**Nuclear Hormone Receptor family#**	**2.7**
nhr-74*	**Nuclear Hormone Receptor family#**	**4.8**

*nhr-113**	Nuclear Hormone Receptor family[#]	4.3
nhr-259	Nuclear Hormone Receptor family	3.3
F21G4.5	F21G4.5	4.0
T16H12.4	General transcription factor IIH subunit 2	4.8
ztf-29	Y66D12A.12	4.4
nhr-244	Nuclear Hormone Receptor family	1.7
nhr-155	Nuclear Hormone Receptor family	5.1
nhr-171	Nuclear Hormone Receptor family	4.8
Gene group 3	*Signaling proteins (enrichment score 0.21)*	
ajm-1	Apical Junction Molecule[§]	1.8
dpy-6	DumPY : shorter than wild-type	3.8
*grd-3**	GRounDhog (hedgehog-like family)[#]	2.5
*grd-14**	GRounDhog (hedgehog-like family)[#]	2.4
*hog-1**	HOG only (Hedgehog Hog domain alone)	3.1
*ldb-1**	LIM domain Binding Protein[Δ]	1.9
*osm-11**	OSMotic avoidance abnormal[§]	1.6
F54D5.5	F54D5.5	2.2
cdr-4	CaDmium Responsive	2.1
T08A11.1	T08A11.1	1.5
B0416.5	Uncharacterized protein	1.5
C35D10.13	C35D10.13	2.7
C44C1.1	C44C1.1	3.3
F10E7.6	F10E7.6	2.6
*Y71H2AL.1**	Y71H2AL.1	1.7
*bus-8**	Bacterially Un-Swollen (M. nematophilum resistant)[#]	2.9
Gene group 4	*Kinases (enrichment score 0.21)*	
*cdk-4**	Cyclin-Dependent Kinase family[§]	3.9
old-1	Overexpression Longevity Determinant	6.7
*old-2**	Overexpression Longevity Determinant	6.7
*pak-1**	P21-Activated Kinase family[Δ]	1.6
*F26E4.5**	F26E4.5	3.5

Table S5 27 seam cell/VPC Wnt regulated genes validated by qPCR. 105 genes (~44%) of 239 putative Wnt target genes were tested by qRT-PCR to validate the directional change observed by microarray, and 27 (26%) genes passed the verification screen (were upregulated ≥1.5 fold in response to Wnt pathway over-activation). Twelve genes (shown in the bottom half of the table) were also downregulated ≥ 1.5 fold in response to Wnt pathway under-activation. *gpd-2* was the reference gene. Biological triplicates of control (*deIs10*) and experimental samples (*deIs10; huIs1* and *deIs10; deIs26*) were analyzed.

Gene WB ID	Gene	fold change Wnt over-activation	fold change Wnt under-activation
WBGene00000626	*col-49*	947.5	1.3
WBGene00012046	*T26E4.4*	190.1	1.6
WBGene00010743	*K10D6.3*	151.0	0.9
WBGene00021997	*Y59E9AR.1*	6.8	1.2
WBGene00010538	*ttr-3*	5.7	1.2
WBGene00000516	*cki-1*	4.5	1.2
WBGene00009590	*ttr-4*	4.5	1.0
WBGene00015769	*C14C11.7*	4.1	1.2
WBGene00010856	*M04C9.1*	3.0	0.8
WBGene00015605	*C08E3.13*	2.8	0.8
WBGene00003102	*mab-5*	2.0	0.9
WBGene00021379	*Y37E11B.7*	1.9	1.1
WBGene00002990	*lin-1*	1.6	0.6
WBGene00021927	*Y55F3AM.10*	1.6	1.1
WBGene00003763	*nlp-25*	1.5	0.7
WBGene00011561	*ttr-15*	1.5	0.8
WBGene00009807	*pepm-1*	10.4	0.3
WBGene00004202	*pry-1*	4.1	0.1
WBGene00008435	*glna-2*	3.8	0.5
WBGene00000072	*add-1*	2.2	0.1
WBGene00001692	*grd-3*	1.8	0.1
WBGene00006660	*twk-5*	1.7	0.5
WBGene00012783	*Y43C5A.3*	1.7	0.4
WBGene00015196	*B0454.5*	1.6	0.2
WBGene00000406	*cdk-4*	1.6	0.4
WBGene00012591	*nspe-1*	1.6	0.3
WBGene00005025	*sqv-7*	1.5	0.5

Table S6 Reduction of function for molting genes *mlt-7* and *mlt-11* causes a reduction in terminal seam cell number. Strains carrying the indicated seam cell marker were grown on bacteria expressing double stranded RNA for each indicated gene and the number of GFP-expressing seam cells per side was scored in young adults. Either larva were grown on bacteria and their F1 progeny were scored as adults ('P0' feeding) or newly hatched L1 larvae were grown on bacteria and the same animals were scored as adults ('L1' feeding). *rrf-3(lf)* renders animals hypersensitive to RNAi. The number of animals scored, average number of seam cells per side, and range of seam cells is indicated.

RNAi	Strain	Method	N	Seam cells number	Seam cells range	P value
vector	*rrf-3(lf); grd-10p::GFP*	P0	30	15.9	15-16	
acn-1	*rrf-3(lf); grd-10p::GFP*	P0	60	14.3	12-19	NS
bli-5	*rrf-3(lf); grd-10p::GFP*	P0	30	14.7	13-16	<.005
fbn-1	*rrf-3(lf); grd-10p::GFP*	P0	30	15.2	14-16	NS
mam-1	*rrf-3(lf); grd-10p::GFP*	P0	30	14.9	13-16	NS
mlt-8	*rrf-3(lf); grd-10p::GFP*	P0	30	14.5	12-17	<.005
mlt-9	*rrf-3(lf); grd-10p::GFP*	P0	30	13.5	11-16	<.001
mlt-10	*rrf-3(lf); grd-10p::GFP*	P0	30	15.4	14-16	NS
nas-36	*rrf-3(lf); grd-10p::GFP*	P0	30	17.1	16-29	<.005
nas-37	*rrf-3(lf); grd-10p::GFP*	P0	30	14.3	11-16	<.005
pan-1	*rrf-3(lf); grd-10p::GFP*	P0	30	13.8	13-16	<.001
ptr-23	*rrf-3(lf); grd-10p::GFP*	P0	30	15.4	13-16	NS
mlt-7	*rrf-3(lf); grd-10::GFP*	P0	90	10.9	3-16	<.001
mlt-7	*wrt-2p::GFP*	P0	90	12.8	8-16	<.001
mlt-7	*scm::GFP*	P0	60	12.3	9-15	<.001
mlt-11	*rrf-3(lf); grd-10p::GFP*	P0	60	10.5	7-13	<.001
mlt-11	*rrf-3(lf); grd-10p::GFP*	L1	60	10.1	6-14	<.001
mlt-11	*wrt-2p::GFP*	P0	60	11.6	8-16	<.001
mlt-11	*wrt-2p::GFP*	L1	90	12.3	8-17	<.001

Table S7 Oligonucleotide sequences

Available for download as an Excel file at www.g3journal.org/lookup/suppl/doi:10.1534/g3.115.017715/-/DC1
