# Supplementary material for: Identification of Wnt Pathway Target Genes Regulating the Division and Differentiation of Larval Seam Cells and Vulval Precursor Cells in Caenorhabditis elegans
Source: G3 (Bethesda). 2015 Jun 5;5(8):1551–66. doi: 10.1534/g3.115.017715 (PMC4528312; doi:10.1534/g3.115.017715)
Supplement: Supporting Information [file supp_g3.115.017715_FigureS1.pdf]

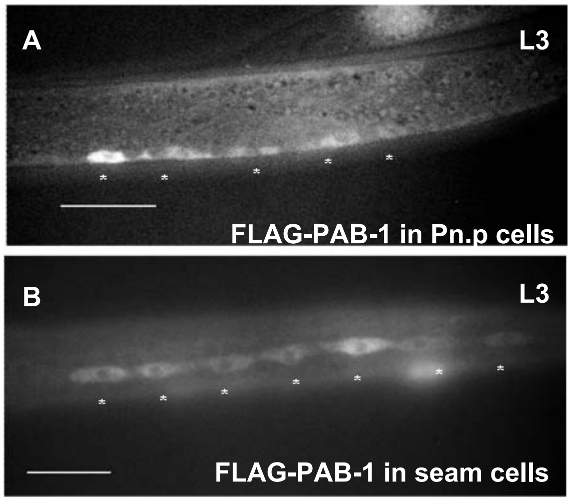

Figure S1 mRNA tagging method requires FLAG-PAB-1 expression in seam cells and VPCs

FLAG-PAB-1 expressed in the L2/L3 worms can be seen in the VPCs (A) and seam cells (B) of the experimental strain *deIs10; scm::gfp* by antibody staining against the FLAG epitope. The asterisks mark the stained cells. Scale bars represent 50µm.
