# Supplementary material for: Identification of Wnt Pathway Target Genes Regulating the Division and Differentiation of Larval Seam Cells and Vulval Precursor Cells in Caenorhabditis elegans
Source: G3 (Bethesda). 2015 Jun 5;5(8):1551–66. doi: 10.1534/g3.115.017715 (PMC4528312; doi:10.1534/g3.115.017715)
Supplement: Supporting Information [file supp_g3.115.017715_TableS1.pdf]

Table S1 239 putative Wnt targets from seam cells and VPCs. Shown are 239 putative Wnt targets identified by microarray analysis: the average fold change and p value in Wnt pathway over-activation conditions is shown for each gene. '*' indicates 24 genes previously known to be expressed in the seam cells (Wormbase release 220). '§' indicates 12 genes previously known to be expressed in the vulval cell lineage (Wormbase release 220) '#' indicates five genes shown to be expressed in seam cells, hypodermal cells and P cells by Jackson et al., 2014.

Gene WB ID	Gene Public Name	p-value Wnt over-activation	fold change Wnt Over-activation
WBGene00000615	*col-38*#	0.000	29.7
WBGene00000626	*col-49*#	0.000	23.5
WBGene00013754	*fbxa-116*	0.002	16.1
WBGene00006509	*tag-164*	0.000	14.6
WBGene00007381	*C06C6.7*	0.000	14.3
WBGene00018295	*oac-29*	0.002	14.2
WBGene00012046	*T26E4.4*#	0.003	13.3
WBGene00014079	*ZK792.7*	0.000	12.8
WBGene00022140	*Y71G12A.4*	0.008	12.1
WBGene00009898	*dod-23*	0.000	11.8
WBGene00013986	*ZK512.7*	0.001	11.2
WBGene00001068	*dpy-6*	0.004	10.7
WBGene00015765	*C14C11.1*	0.000	10.3
WBGene00022589	*ZC317.6*	0.000	10.3
WBGene00008211	*C49F5.7*	0.002	10.3
WBGene00016058	*nspd-3*	0.001	10.0
WBGene00012783	*Y43C5A.3*	0.001	10.0
WBGene00003763	*nlp-25*	0.017	10.0
WBGene00044630	*bus-17*	0.007	9.6
WBGene00017485	*F15E6.4*	0.001	9.4
WBGene00017488	*dct-7*	0.031	8.7
WBGene00016683	*C45G9.12*	0.004	8.5
WBGene00011226	*R11.1*	0.001	7.8
WBGene00011624	*T08G5.3*	0.004	7.8
WBGene00021379	*Y37E11B.7*	0.034	7.7
WBGene00012591	*nspe-1*	0.022	7.5
WBGene00015182	*B0416.7*	0.002	7.4
WBGene00001691	*grd-2*	0.004	6.9
WBGene00003862	*old-1*	0.004	6.7
WBGene00003863	*old-2*	0.004	6.7
WBGene00018380	*F43C11.3*	0.036	6.6
WBGene00021731	*Y49G5A.1*	0.000	6.3
WBGene00000647	*col-71*#	0.001	6.2
WBGene00020033	*R12E2.7*§*	0.012	6.2
WBGene00017759	*F23H11.3*	0.002	6.0
WBGene00008472	*E03H4.4*	0.000	6.0
WBGene00013514	*Y73F4A.1*	0.019	6.0

**WBGene00018744**	***F53B3.6***	**0.008**	**5.9**
**WBGene00022024**	***Y64H9A.2***	**0.000**	**5.9**
**WBGene00009130**	***F25H5.8***	**0.012**	**5.8**
**WBGene00000656**	***col-80***	**0.007**	**5.8**
**WBGene00001079**	***dpy-20***	**0.002**	**5.6**
**WBGene00007297**	***C04F12.1***	**0.004**	**5.5**
**WBGene00050896**	***ttr-56***	**0.000**	**5.4**
**WBGene00010856**	***M04C9.1***	**0.001**	**5.4**
**WBGene00010743**	***K10D6.3***	**0.012**	**5.2**
**WBGene00001882**	***his-8***	**0.001**	**5.2**
**WBGene00001894**	***his-20***	**0.001**	**5.2**
**WBGene00001896**	***his-22***	**0.001**	**5.2**
**WBGene00001926**	***his-52***	**0.001**	**5.2**
**WBGene00011077**	***R07B1.5***	**0.007**	**5.2**
**WBGene00004394**	***rol-1***	**0.001**	**5.2**
**WBGene00021427**	***Y38F2AR.9***	**0.006**	**5.2**
**WBGene00015758**	***nhr-155***	**0.001**	**5.1**
**WBGene00000930**	***dao-4*** [#]	**0.011**	**5.0**
**WBGene00018219**	***F40A3.4***	**0.003**	**5.0**
**WBGene00015865**	***C16E9.1***	**0.004**	**4.9**
**WBGene00016926**	***nhr-171***	**0.005**	**4.8**
**WBGene00011948**	***T23F1.5***	**0.000**	**4.8**
**WBGene00003664**	***nhr-74****	**0.004**	**4.8**
**WBGene00011814**	***T16H12.4***	**0.011**	**4.8**
**WBGene00045387**	***K09H9.8***	**0.004**	**4.7**
**WBGene00021994**	***Y59E9AL.4***	**0.005**	**4.7**
**WBGene00011533**	***T06E4.5***	**0.010**	**4.6**
**WBGene00020380**	***T09B4.6***	**0.006**	**4.5**
**WBGene00020769**	***T24D8.6***	**0.020**	**4.5**
**WBGene00021605**	***Y46H3C.5***	**0.013**	**4.5**
**WBGene00015172**	***B0410.3***	**0.001**	**4.5**
**WBGene00014003**	***ZK593.3***	**0.050**	**4.5**
**WBGene00011023**	***R05A10.6***	**0.014**	**4.5**
**WBGene00015769**	***C14C11.7***	**0.001**	**4.5**
**WBGene00013438**	***ztf-29***	**0.030**	**4.4**
**WBGene00003703**	***nhr-113****	**0.019**	**4.3**
**WBGene00015340**	***C02E7.7***	**0.000**	**4.3**
**WBGene00006534**	***tba-8****	**0.001**	**4.3**
**WBGene00012594**	***nspe-5***	**0.000**	**4.3**
**WBGene00004202**	***pry-1*** [§*]	**0.018**	**4.2**
**WBGene00017436**	***F13H8.3***	**0.029**	**4.1**
**WBGene00020613**	***T20D4.7***	**0.036**	**4.1**
**WBGene00000493**	***che-14*** [§*]	**0.037**	**4.1**
**WBGene00022816**	***fbn-1***	**0.027**	**4.1**
**WBGene00043147**	***nspd-6***	**0.004**	**4.1**
**WBGene00015339**	***C02E7.6***	**0.000**	**4.1**
**WBGene00009026**	***F21G4.5***	**0.008**	**4.0**

**WBGene00000406**	***cdk-4*** [§*]	**0.007**	**3.9**
**WBGene00005672**	***sru-9***	**0.000**	**3.9**
**WBGene00011594**	***T07G12.3***	**0.033**	**3.9**
**WBGene00001885**	***his-11***	**0.005**	**3.9**
**WBGene00001889**	***his-15***	**0.005**	**3.9**
**WBGene00001918**	***his-44****	**0.005**	**3.9**
**WBGene00019306**	***K02E7.1***	**0.043**	**3.9**
**WBGene00018416**	***retr-1***	**0.007**	**3.9**
**WBGene00009787**	***F46F2.3***	**0.018**	**3.8**
**WBGene00000516**	***cki-1*** [§*]	**0.007**	**3.8**
**WBGene00015196**	***B0454.5***	**0.011**	**3.8**
**WBGene00000072**	***add-1****	**0.009**	**3.7**
**WBGene00011033**	***R05D11.5***	**0.000**	**3.7**
**WBGene00017297**	***F09E10.7***	**0.002**	**3.7**
**WBGene00000653**	***col-77***	**0.001**	**3.7**
**WBGene00013661**	***Y105C5B.23***	**0.031**	**3.7**
**WBGene00001878**	***his-4***	**0.003**	**3.7**
**WBGene00002393**	***lpr-1***	**0.020**	**3.6**
**WBGene00017799**	***F25G6.8***	**0.015**	**3.6**
**WBGene00012324**	***rhy-1***	**0.029**	**3.6**
**WBGene00009590**	***ttr-4***	**0.012**	**3.6**
**WBGene00010538**	***ttr-3***	**0.012**	**3.6**
**WBGene00015051**	***B0218.7***	**0.000**	**3.6**
**WBGene00006367**	***sym-2***	**0.001**	**3.6**
**WBGene00003446**	***msp-53***	**0.002**	**3.5**
**WBGene00006539**	***tbb-6***	**0.000**	**3.5**
**WBGene00020873**	***T28A11.6***	**0.027**	**3.5**
**WBGene00011665**	***T09F5.1***	**0.010**	**3.5**
**WBGene00009160**	***F26E4.5***	**0.018**	**3.5**
**WBGene00000711**	***col-138***	**0.000**	**3.4**
**WBGene00011398**	***qdpr-1***	**0.021**	**3.4**
**WBGene00017783**	***F25E5.2***	**0.008**	**3.4**
**WBGene00021997**	***Y59E9AR.1***	**0.001**	**3.4**
**WBGene00022002**	***Y59E9AR.7***	**0.001**	**3.4**
**WBGene00021927**	***Y55F3AM.10***	**0.001**	**3.4**
**WBGene00016919**	***C54E4.4***	**0.025**	**3.4**
**WBGene00015442**	***C04F1.1***	**0.050**	**3.4**
**WBGene00016641**	***C44C1.1***	**0.014**	**3.3**
**WBGene00007770**	***nhr-259***	**0.038**	**3.3**
**WBGene00018689**	***F52D2.7***	**0.001**	**3.3**
**WBGene00015987**	***C18G1.9***	**0.001**	**3.3**
**WBGene00012323**	***oac-54***	**0.032**	**3.3**
**WBGene00018702**	***F52E4.5***	**0.002**	**3.2**
**WBGene00001984**	***hog-1***	**0.018**	**3.1**
**WBGene00019234**	***ugt-8***	**0.011**	**3.1**

**WBGene00000596**	***col-7****	**0.010**	**3.1**
**WBGene00000638**	***col-62***	**0.010**	**3.1**
**WBGene00003102**	***mab-5*** [§*]	**0.013**	**3.1**
**WBGene00008290**	***C54C8.2****	**0.033**	**3.1**
**WBGene00020216**	***trap-2***	**0.031**	**3.1**
**WBGene00020445**	***T12B3.2***	**0.008**	**3.1**
**WBGene00015950**	***C18A11.4***	**0.000**	**3.1**
**WBGene00010225**	***ttr-31***	**0.025**	**3.0**
**WBGene00010800**	***srsx-37***	**0.009**	**3.0**
**WBGene00012055**	***srsx-36***	**0.009**	**3.0**
**WBGene00016192**	***C28H8.1***	**0.002**	**3.0**
**WBGene00000734**	***col-161***	**0.008**	**3.0**
**WBGene00000735**	***col-162***	**0.008**	**3.0**
**WBGene00001480**	***fmo-5***	**0.015**	**3.0**
**WBGene00014162**	***ZK938.6***	**0.006**	**3.0**
**WBGene00016294**	***C31H2.4***	**0.008**	**2.9**
**WBGene00044623**	***bus-8****	**0.013**	**2.9**
**WBGene00015453**	***C04G6.2***	**0.007**	**2.9**
**WBGene00077489**	***C04G6.13***	**0.007**	**2.9**
**WBGene00003444**	***msp-51***	**0.026**	**2.9**
**WBGene00003448**	***msp-55***	**0.026**	**2.9**
**WBGene00003450**	***msp-57***	**0.026**	**2.9**
**WBGene00011434**	***T04D3.5***	**0.016**	**2.8**
**WBGene00006660**	***twk-5***	**0.015**	**2.8**
**WBGene00000969**	***dhs-5***	**0.013**	**2.8**
**WBGene00001253**	***elt-6*** [§]	**0.011**	**2.8**
**WBGene00021486**	***lbp-9***	**0.001**	**2.8**
**WBGene00010742**	***K10D6.2***	**0.004**	**2.7**
**WBGene00016449**	***C35D10.13***	**0.002**	**2.7**
**WBGene00003622**	***nhr-23***	**0.002**	**2.7**
**WBGene00000720**	***col-147***	**0.050**	**2.7**
**WBGene00008435**	***glna-2***	**0.022**	**2.6**
**WBGene00016011**	***C23G10.2***	**0.007**	**2.6**
**WBGene00012165**	***cutl-6***	**0.049**	**2.6**
**WBGene00017348**	***F10E7.6***	**0.036**	**2.6**
**WBGene00009657**	***F43G6.4***	**0.023**	**2.6**
**WBGene00001692**	***grd-3****	**0.008**	**2.5**
**WBGene00012420**	***Y7A9D.1***	**0.007**	**2.5**
**WBGene00009489**	***F36G9.13***	**0.001**	**2.5**
**WBGene00009675**	***F44A6.3***	**0.034**	**2.5**
**WBGene00002070**	***ile-1***	**0.004**	**2.5**
**WBGene00000990**	***dhs-27***	**0.016**	**2.5**
**WBGene00016346**	***fbxa-120***	**0.006**	**2.5**
**WBGene00018252**	***F40H3.6***	**0.003**	**2.5**
**WBGene00012364**	***W09D12.1***	**0.009**	**2.5**

**WBGene00001703**	***grd-14****	**0.002**	**2.4**
**WBGene00010289**	***F58H1.6***	**0.015**	**2.4**
**WBGene00021845**	***rpb-7***	**0.000**	**2.4**
**WBGene00007592**	***C14H10.2***	**0.015**	**2.4**
**WBGene00012443**	***Y15E3A.4***	**0.010**	**2.3**
**WBGene00009807**	***pepm-1***	**0.004**	**2.3**
**WBGene00015593**	***C08E3.1***	**0.036**	**2.3**
**WBGene00015605**	***C08E3.13***	**0.036**	**2.3**
**WBGene00000601**	***col-12***	**0.028**	**2.3**
**WBGene00003611**	***nhr-12***	**0.015**	**2.3**
**WBGene00005025**	***sqv-7***§*	**0.007**	**2.2**
**WBGene00017082**	***DC2.5***	**0.004**	**2.2**
**WBGene00010051**	***F54D5.5***	**0.018**	**2.2**
**WBGene00011890**	***T21C9.1***	**0.005**	**2.2**
**WBGene00007925**	***C34C12.6***	**0.022**	**2.1**
**WBGene00006476**	***rhgf-2***	**0.009**	**2.1**
**WBGene00000532**	***clh-5***	**0.031**	**2.1**
**WBGene00000602**	***col-13***	**0.036**	**2.1**
**WBGene00019666**	***sago-1***§*	**0.024**	**2.1**
**WBGene00016929**	***C54F6.12***	**0.024**	**2.1**
**WBGene00019331**	***dos-3***	**0.008**	**2.1**
**WBGene00004272**	***rab-8***	**0.045**	**2.1**
**WBGene00044349**	***Y71G12B.32***	**0.000**	**2.1**
**WBGene00000729**	***col-156***	**0.027**	**2.1**
**WBGene00013981**	***ZK507.6***	**0.036**	**2.1**
**WBGene00010470**	***cdr-4***	**0.027**	**2.1**
**WBGene00013292**	***Y57G11A.4***	**0.017**	**2.0**
**WBGene00011561**	***ttr-15***	**0.009**	**2.0**
**WBGene00001073**	***dpy-11***	**0.026**	**2.0**
**WBGene00021789**	***nol-6***	**0.017**	**2.0**
**WBGene00010523**	***K03B8.4***	**0.040**	**2.0**
**WBGene00001971**	***hmg-1.1***§*	**0.022**	**2.0**
**WBGene00019826**	***R02E4.1***	**0.004**	**2.0**
**WBGene00002990**	***lin-1***	**0.038**	**1.9**
**WBGene00018488**	***acs-1***	**0.048**	**1.9**
**WBGene00004164**	***pqn-83***	**0.015**	**1.9**
**WBGene00004702**	***rsp-5***	**0.004**	**1.9**
**WBGene00002261**	***ldb-1***	**0.047**	**1.9**
**WBGene00006543**	***tbx-2***	**0.039**	**1.9**
**WBGene00008571**	***F08B12.1***	**0.040**	**1.9**
**WBGene00020109**	***R151.6***	**0.013**	**1.9**
**WBGene00012186**	***mlt-11****	**0.043**	**1.8**
**WBGene00020263**	***T05E8.3***	**0.010**	**1.8**
**WBGene00011731**	***acbp-5***	**0.037**	**1.8**
**WBGene00018341**	***F42A10.5***	**0.049**	**1.8**

WBGene00000100	*ajm-1*[§*]	0.026	1.8
WBGene00000730	*col-157*	0.023	1.8
WBGene00000728	*col-155*	0.030	1.8
WBGene00004701	*rsp-4**	0.006	1.7
WBGene00000727	*col-154*	0.029	1.7
WBGene00022164	*Y71H2AL.1*	0.042	1.7
WBGene00014186	*nhr-244*	0.019	1.7
WBGene00002051	*ifa-3*	0.004	1.7
WBGene00019850	*srt-18*	0.019	1.7
WBGene00009381	*F34H10.4*	0.023	1.7
WBGene00019237	*H24G06.1*	0.021	1.7
WBGene00001928	*his-54*	0.042	1.7
WBGene00003911	*pak-1*	0.025	1.6
WBGene00010317	*idh-1*[§*]	0.044	1.6
WBGene00003891	*osm-11*[§*]	0.047	1.6
WBGene00015757	*C14C6.3*	0.009	1.6
WBGene00014098	*ZK836.2*	0.022	1.6
WBGene00011604	*T08A11.1*	0.029	1.5
WBGene00015181	*B0416.5*	0.003	1.5
WBGene00002196	*kin-10*	0.124	1.5
