# Supplementary material for: Identification of Wnt Pathway Target Genes Regulating the Division and Differentiation of Larval Seam Cells and Vulval Precursor Cells in Caenorhabditis elegans
Source: G3 (Bethesda). 2015 Jun 5;5(8):1551–66. doi: 10.1534/g3.115.017715 (PMC4528312; doi:10.1534/g3.115.017715)
Supplement: Supporting Information [file supp_g3.115.017715_TableS2.pdf]

Table S2 24 putative Wnt target genes in common between Jackson et al., 2014 and this work. Of the 239 putative Wnt pathway regulated genes from seam cells and VPCs identified here by mRNA tagging, 24 genes were also identified by Jackson et al., 2014 in a non-tissue specific analysis of Wnt upregulated genes.

Gene WB ID	Gene
WBGene00000615	*col-38*
WBGene00000626	*col-49*
WBGene00000647	*col-71*
WBGene00000930	*dao-4*
WBGene00001984	*hog-1*
WBGene00004202	*pry-1*
WBGene00006509	*tag-164*
WBGene00006539	*tbb-6*
WBGene00008472	*E03H4.4*
WBGene00009898	*dod-23*
WBGene00010470	*cdr-4*
WBGene00011077	*R07B1.5*
WBGene00011594	*T07G12.3*
WBGene00011624	*T08G5.3*
WBGene00012046	*T26E4.4*
WBGene00012783	*Y43C5A.3*
WBGene00013754	*fbxa-116*
WBGene00015442	*C04F1.1*
WBGene00016058	*nspd-3*
WBGene00017485	*F15E6.4*
WBGene00018295	*oac-29*
WBGene00018416	*retr-1*
WBGene00019234	*ugt-8*
WBGene00043147	*nspd-6*
