# Supplementary material for: Identification of Wnt Pathway Target Genes Regulating the Division and Differentiation of Larval Seam Cells and Vulval Precursor Cells in Caenorhabditis elegans
Source: G3 (Bethesda). 2015 Jun 5;5(8):1551–66. doi: 10.1534/g3.115.017715 (PMC4528312; doi:10.1534/g3.115.017715)
Supplement: Supporting Information [file supp_g3.115.017715_TableS3.pdf]

Table S3 Genes in common between van der Bent et al., 2014 and this work. Of the 239 putative Wnt pathway regulated genes from seam cells and VPCs identified here by mRNA tagging, 35 genes were also identified by van der Bent et al., 2014 as genes that were differentially expressed between a wild type strain and a strain carrying a loss-of-function mutation in *bar-1*. The third column indicates whether expression of the gene was decreased or increased in the *bar-1(ga80)* strain relative to wild type. Genes with an asterisk (*) were identified as downregulated upon loss of *bar-1* in van der Bent et al. 2014 and upregulated upon expression of an activated BAR-1 protein in this work and in Jackson et al . 2014.

Gene WB ID	Gene	*bar-1(ga80)* expression
WBGene00000493	*che-14*	decreased
WBGene00000615	*col-38**	decreased
WBGene00000626	*col-49**	decreased
WBGene00000647	*col-71**	decreased
WBGene00000711	*col-138**	decreased
WBGene00000735	*col-162*	decreased
WBGene00000930	*dao-4**	decreased
WBGene00001073	*dpy-11**	decreased
WBGene00001691	*grd-2**	decreased
WBGene00001885	*his-11*	decreased
WBGene00001984	*hog-1**	decreased
WBGene00002393	*lpr-1*	decreased
WBGene00004394	*rol-1*	decreased
WBGene00011077	*R07B1.5**	decreased
WBGene00011594	*T07G12.3**	decreased
WBGene00011665	*T09F5.1*	decreased
WBGene00012186	*mlt-11*	decreased
WBGene00013514	*Y73F4A.1*	decreased
WBGene00015172	*B0410.3*	decreased
WBGene00015442	*C04F1.1**	decreased
WBGene00015453	*C04G6.2*	decreased
WBGene00015950	*C18A11.4**	decreased
WBGene00017783	*F25E5.2**	decreased
WBGene00022024	*Y64H9A.2*	decreased
WBGene00077489	*C04G6.13*	decreased
WBGene00000720	*col-147*	increased
WBGene00006539	*tbb-6*	Increased
WBGene00007297	*C04F12.1*	Increased
WBGene00010051	*F54D5.5*	Increased
WBGene00011434	*T04D3.5*	Increased
WBGene00011561	*ttr-15*	Increased
WBGene00012783	*Y43C5A.3*	Increased
WBGene00015865	*C16E9.1*	Increased
WBGene00016919	*C54E4.4*	Increased
WBGene00021379	*Y37E11B.7*	increased
