# Supplementary material for: Identification of Wnt Pathway Target Genes Regulating the Division and Differentiation of Larval Seam Cells and Vulval Precursor Cells in Caenorhabditis elegans
Source: G3 (Bethesda). 2015 Jun 5;5(8):1551–66. doi: 10.1534/g3.115.017715 (PMC4528312; doi:10.1534/g3.115.017715)
Supplement: Supporting Information [file supp_g3.115.017715_TableS4.pdf]

Table S4 DAVID analysis identifies four functionally related groups among cell type specific Wnt targets. DAVID analysis of the seam cell/VPC Wnt regulated genes identified four functionally related gene groups. The microarray fold change in the Wnt over-activation condition for each gene is indicated. The enrichment score for each gene group is indicated in parenthesis (column 2). The enrichment score is the geometric mean of the modified Fisher's exact p-value (EASE score) of each gene in a given gene group. A higher enrichment score indicates a higher rank in functional importance (Huang et al. 2009a, 2009b). Of these 57 genes grouped by DAVID, seven genes are expressed in seam (indicated by '#', column 2), two genes in the vulva (indicated by '∆') and five in the seam and vulva (indicated by '§') (Wormbase release 220). '*' indicates genes in this list that were characterized further in this work.

Gene	Gene description	fold change Wnt over-activation
Gene group 1	***Structural constituents of cuticle (enrichment score 7.3)***	
che-14*	**abnormal CHEmotaxis§**	**4.1**
col-7	**COLlagen#**	**3.1**
col-12	**COLlagen**	**2.3**
col-13	**COLlagen**	**2.1**
col-38	**COLlagen**	**29.7**
col-49*	**COLlagen**	**23.5**
col-62	**COLlagen**	**3.1**
col-71	**COLlagen**	**6.2**
col-77	**COLlagen**	**3.7**
col-80	**COLlagen**	**5.8**
col-138	**COLlagen**	**3.4**
col-147	**COLlagen**	**2.7**
col-154	**COLlagen**	**1.7**
col-155	**COLlagen**	**1.8**
col-156	**COLlagen**	**2.1**
col-157	**COLlagen**	**1.8**
col-161	**COLlagen**	**3.0**
col-162	**COLlagen**	**3.0**
pqn-83	**Prion-like-(Q/N-rich)-domain protein**	**1.9**
rol-1	**ROLler/collagen**	**5.2**
F54D5.5	**F54D5.5**	**2.2**
K03B8.4	**K03B8.4**	**2.0**
T04D3.5	**T04D3.5**	**2.8**
T06E4.5	**T06E4.5**	**4.6**
C44C1.1	**C44C1.1**	**3.3**
Gene group 2	***Transcription factors (enrichment score 0.64)***	
dpy-20	**DumPY**	**5.6**
elt-6*	**Erythroid-Like Transcription factor family§**	**2.8**
nhr-12	**Nuclear Hormone Receptor family**	**2.3**
nhr-23*	**Nuclear Hormone Receptor family#**	**2.7**
nhr-74*	**Nuclear Hormone Receptor family#**	**4.8**

*nhr-113**	Nuclear Hormone Receptor family[#]	4.3
nhr-259	Nuclear Hormone Receptor family	3.3
F21G4.5	F21G4.5	4.0
T16H12.4	General transcription factor IIH subunit 2	4.8
ztf-29	Y66D12A.12	4.4
nhr-244	Nuclear Hormone Receptor family	1.7
nhr-155	Nuclear Hormone Receptor family	5.1
nhr-171	Nuclear Hormone Receptor family	4.8
Gene group 3	*Signaling proteins (enrichment score 0.21)*	
ajm-1	Apical Junction Molecule[§]	1.8
dpy-6	DumPY : shorter than wild-type	3.8
*grd-3**	GRounDhog (hedgehog-like family)[#]	2.5
*grd-14**	GRounDhog (hedgehog-like family)[#]	2.4
*hog-1**	HOG only (Hedgehog Hog domain alone)	3.1
*ldb-1**	LIM domain Binding Protein[Δ]	1.9
*osm-11**	OSMotic avoidance abnormal[§]	1.6
F54D5.5	F54D5.5	2.2
cdr-4	CaDmium Responsive	2.1
T08A11.1	T08A11.1	1.5
B0416.5	Uncharacterized protein	1.5
C35D10.13	C35D10.13	2.7
C44C1.1	C44C1.1	3.3
F10E7.6	F10E7.6	2.6
*Y71H2AL.1**	Y71H2AL.1	1.7
*bus-8**	Bacterially Un-Swollen (M. nematophilum resistant)[#]	2.9
Gene group 4	*Kinases (enrichment score 0.21)*	
*cdk-4**	Cyclin-Dependent Kinase family[§]	3.9
old-1	Overexpression Longevity Determinant	6.7
*old-2**	Overexpression Longevity Determinant	6.7
*pak-1**	P21-Activated Kinase family[Δ]	1.6
*F26E4.5**	F26E4.5	3.5
