# Supplementary material for: Identification of Wnt Pathway Target Genes Regulating the Division and Differentiation of Larval Seam Cells and Vulval Precursor Cells in Caenorhabditis elegans
Source: G3 (Bethesda). 2015 Jun 5;5(8):1551–66. doi: 10.1534/g3.115.017715 (PMC4528312; doi:10.1534/g3.115.017715)
Supplement: Supporting Information [file supp_g3.115.017715_TableS5.pdf]

Table S5 27 seam cell/VPC Wnt regulated genes validated by qPCR. 105 genes (~44%) of 239 putative Wnt target genes were tested by qRT-PCR to validate the directional change observed by microarray, and 27 (26%) genes passed the verification screen (were upregulated ≥1.5 fold in response to Wnt pathway over-activation). Twelve genes (shown in the bottom half of the table) were also downregulated ≥ 1.5 fold in response to Wnt pathway under-activation. *gpd-2* was the reference gene. Biological triplicates of control (*deIs10*) and experimental samples (*deIs10; huIs1* and *deIs10; deIs26*) were analyzed.

Gene WB ID	Gene	fold change Wnt over-activation	fold change Wnt under-activation
WBGene00000626	*col-49*	947.5	1.3
WBGene00012046	*T26E4.4*	190.1	1.6
WBGene00010743	*K10D6.3*	151.0	0.9
WBGene00021997	*Y59E9AR.1*	6.8	1.2
WBGene00010538	*ttr-3*	5.7	1.2
WBGene00000516	*cki-1*	4.5	1.2
WBGene00009590	*ttr-4*	4.5	1.0
WBGene00015769	*C14C11.7*	4.1	1.2
WBGene00010856	*M04C9.1*	3.0	0.8
WBGene00015605	*C08E3.13*	2.8	0.8
WBGene00003102	*mab-5*	2.0	0.9
WBGene00021379	*Y37E11B.7*	1.9	1.1
WBGene00002990	*lin-1*	1.6	0.6
WBGene00021927	*Y55F3AM.10*	1.6	1.1
WBGene00003763	*nlp-25*	1.5	0.7
WBGene00011561	*ttr-15*	1.5	0.8
WBGene00009807	*pepm-1*	10.4	0.3
WBGene00004202	*pry-1*	4.1	0.1
WBGene00008435	*glna-2*	3.8	0.5
WBGene00000072	*add-1*	2.2	0.1
WBGene00001692	*grd-3*	1.8	0.1
WBGene00006660	*twk-5*	1.7	0.5
WBGene00012783	*Y43C5A.3*	1.7	0.4
WBGene00015196	*B0454.5*	1.6	0.2
WBGene00000406	*cdk-4*	1.6	0.4
WBGene00012591	*nspe-1*	1.6	0.3
WBGene00005025	*sqv-7*	1.5	0.5
