# Supplementary material for: Identification of Wnt Pathway Target Genes Regulating the Division and Differentiation of Larval Seam Cells and Vulval Precursor Cells in Caenorhabditis elegans
Source: G3 (Bethesda). 2015 Jun 5;5(8):1551–66. doi: 10.1534/g3.115.017715 (PMC4528312; doi:10.1534/g3.115.017715)
Supplement: Supporting Information [file supp_g3.115.017715_FigureS2.pdf]

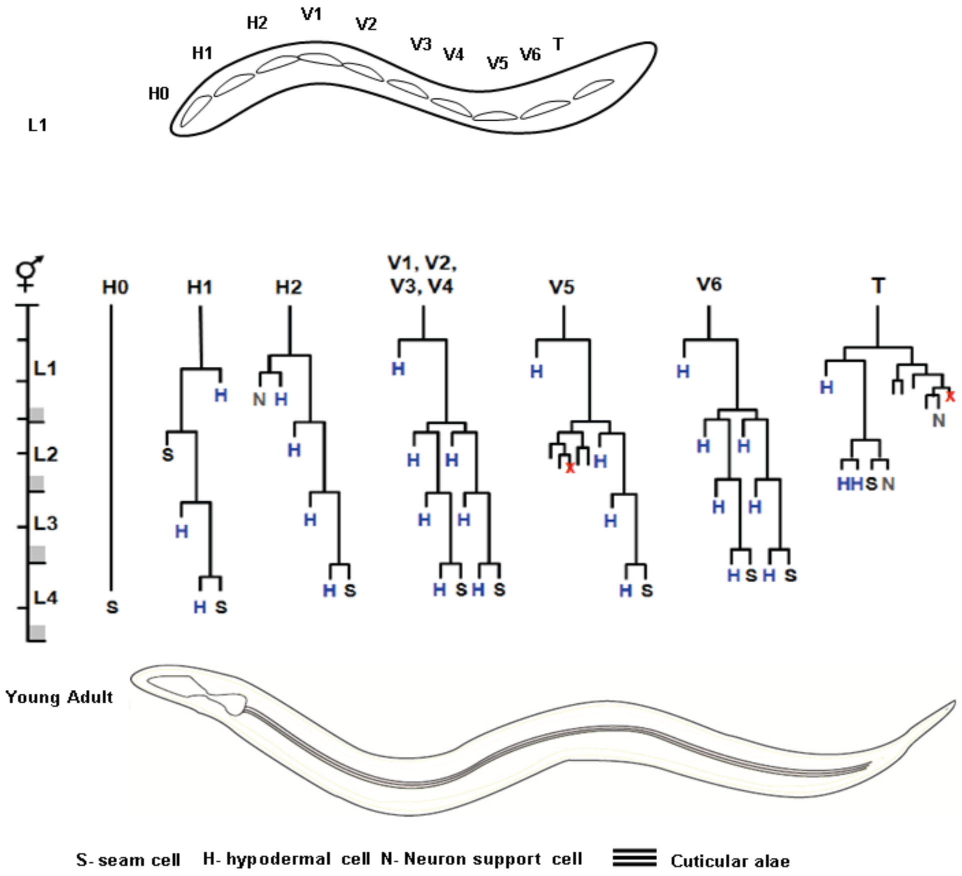

Figure S2 Lineage of larval seam cells

An L1 worm has 10 seam cells. At L2, six seam cells undergo a proliferative division to increase the number of seam cells to 16. In each larval stage, most seam cells divide asymmetrically to maintain their population as well as contribute to the hypodermal syncytium and to other neuronal support structures. After their final division, seam cells differentiate and undergo homotypic fusion to form a single-celled syncytium which secretes a specialized cuticular structure called alae.
