# Supplementary material for: Identification of Wnt Pathway Target Genes Regulating the Division and Differentiation of Larval Seam Cells and Vulval Precursor Cells in Caenorhabditis elegans
Source: G3 (Bethesda). 2015 Jun 5;5(8):1551–66. doi: 10.1534/g3.115.017715 (PMC4528312; doi:10.1534/g3.115.017715)
Supplement: Supporting Information [file supp_g3.115.017715_TableS6.pdf]

Table S6 Reduction of function for molting genes *mlt-7* and *mlt-11* causes a reduction in terminal seam cell number. Strains carrying the indicated seam cell marker were grown on bacteria expressing double stranded RNA for each indicated gene and the number of GFP-expressing seam cells per side was scored in young adults. Either larva were grown on bacteria and their F1 progeny were scored as adults ('P0' feeding) or newly hatched L1 larvae were grown on bacteria and the same animals were scored as adults ('L1' feeding). *rrf-3(lf)* renders animals hypersensitive to RNAi. The number of animals scored, average number of seam cells per side, and range of seam cells is indicated.

RNAi	Strain	Method	N	Seam cells number	Seam cells range	P value
vector	*rrf-3(lf); grd-10p::GFP*	P0	30	15.9	15-16	
acn-1	*rrf-3(lf); grd-10p::GFP*	P0	60	14.3	12-19	NS
bli-5	*rrf-3(lf); grd-10p::GFP*	P0	30	14.7	13-16	<.005
fbn-1	*rrf-3(lf); grd-10p::GFP*	P0	30	15.2	14-16	NS
mam-1	*rrf-3(lf); grd-10p::GFP*	P0	30	14.9	13-16	NS
mlt-8	*rrf-3(lf); grd-10p::GFP*	P0	30	14.5	12-17	<.005
mlt-9	*rrf-3(lf); grd-10p::GFP*	P0	30	13.5	11-16	<.001
mlt-10	*rrf-3(lf); grd-10p::GFP*	P0	30	15.4	14-16	NS
nas-36	*rrf-3(lf); grd-10p::GFP*	P0	30	17.1	16-29	<.005
nas-37	*rrf-3(lf); grd-10p::GFP*	P0	30	14.3	11-16	<.005
pan-1	*rrf-3(lf); grd-10p::GFP*	P0	30	13.8	13-16	<.001
ptr-23	*rrf-3(lf); grd-10p::GFP*	P0	30	15.4	13-16	NS
mlt-7	*rrf-3(lf); grd-10::GFP*	P0	90	10.9	3-16	<.001
mlt-7	*wrt-2p::GFP*	P0	90	12.8	8-16	<.001
mlt-7	*scm::GFP*	P0	60	12.3	9-15	<.001
mlt-11	*rrf-3(lf); grd-10p::GFP*	P0	60	10.5	7-13	<.001
mlt-11	*rrf-3(lf); grd-10p::GFP*	L1	60	10.1	6-14	<.001
mlt-11	*wrt-2p::GFP*	P0	60	11.6	8-16	<.001
mlt-11	*wrt-2p::GFP*	L1	90	12.3	8-17	<.001
